# Adverse outcome pathways for ionizing radiation and breast cancer involve direct and indirect DNA damage, oxidative stress, inflammation, genomic instability, and interaction with hormonal regulation of the breast

**DOI:** 10.1007/s00204-020-02752-z

**Published:** 2020-05-13

**Authors:** Jessica S. Helm, Ruthann A. Rudel

**Affiliations:** Silent Spring Institute, 320 Nevada Street, Suite 302, Newton, MA 02460 USA

**Keywords:** Ionizing radiation, Breast carcinogenesis, DNA damage, Inflammation, Oxidative stress, Cell proliferation, Genomic instability, Epigenetics, Hormones, Characteristics of carcinogens, Adverse outcome pathway, Mechanisms, Key events, In vitro assays, In vivo assays

## Abstract

Knowledge about established breast carcinogens can support improved and modernized toxicological testing methods by identifying key mechanistic events. Ionizing radiation (IR) increases the risk of breast cancer, especially for women and for exposure at younger ages, and evidence overall supports a linear dose–response relationship. We used the Adverse Outcome Pathway (AOP) framework to outline and evaluate the evidence linking ionizing radiation with breast cancer from molecular initiating events to the adverse outcome through intermediate key events, creating a qualitative AOP. We identified key events based on review articles, searched PubMed for recent literature on key events and IR, and identified additional papers using references. We manually curated publications and evaluated data quality. Ionizing radiation directly and indirectly causes DNA damage and increases production of reactive oxygen and nitrogen species (RONS). RONS lead to DNA damage and epigenetic changes leading to mutations and genomic instability (GI). Proliferation amplifies the effects of DNA damage and mutations leading to the AO of breast cancer. Separately, RONS and DNA damage also increase inflammation. Inflammation contributes to direct and indirect effects (effects in cells not directly reached by IR) via positive feedback to RONS and DNA damage, and separately increases proliferation and breast cancer through pro-carcinogenic effects on cells and tissue. For example, gene expression changes alter inflammatory mediators, resulting in improved survival and growth of cancer cells and a more hospitable tissue environment. All of these events overlap at multiple points with events characteristic of “background” induction of breast carcinogenesis, including hormone-responsive proliferation, oxidative activity, and DNA damage. These overlaps make the breast particularly susceptible to ionizing radiation and reinforce that these biological activities are important characteristics of carcinogens. Agents that increase these biological processes should be considered potential breast carcinogens, and predictive methods are needed to identify chemicals that increase these processes. Techniques are available to measure RONS, DNA damage and mutation, cell proliferation, and some inflammatory proteins or processes. Improved assays are needed to measure GI and chronic inflammation, as well as the interaction with hormonally driven development and proliferation. Several methods measure diverse epigenetic changes, but it is not clear which changes are relevant to breast cancer. In addition, most toxicological assays are not conducted in mammary tissue, and so it is a priority to evaluate if results from other tissues are generalizable to breast, or to conduct assays in breast tissue. Developing and applying these assays to identify exposures of concern will facilitate efforts to reduce subsequent breast cancer risk.

**Electronic supplementary material:**

The online version of this article (10.1007/s00204-020-02752-z) contains supplementary material, which is available to authorized users.

## Introduction

Breast cancer imposes a significant burden on women worldwide and is an important focus for prevention. It is the most common invasive cancer in women with the highest rates found in North America and Europe (Ervik et al. 2016), and incidence is increasing globally (Forouzanfar et al. 2011). Around 60% of all cancers are related to non-heritable factors (Colditz and Wei 2012; Moller et al. 2016; Ronckers et al. 2005), so significant opportunities exist to reduce risk. Understanding breast carcinogenesis from an established breast carcinogen will help to identify early effect markers that can be used in screening chemicals and drugs to identify other breast carcinogens, prevent exposures, and reduce future breast cancers.

Ionizing radiation (IR) is a well-studied carcinogen that increases the risk of breast cancer in people (Bijwaard et al. 2010; Eidemuller et al. 2015; Henderson et al. 2010; Little and McElvenny 2017; Ma et al. 2008; Moskowitz et al. 2014; Neta et al. 2012) and mammary gland tumors in rodents (Imaoka et al. 2009; Rivina et al. 2016; Russo 2015; Wagner 2004), so we selected it for detailed analysis using the Adverse Outcome Pathway (AOP) framework. AOPs provide a structure and methodology for the systematic organization of mechanistic data into a series of discrete measurable events leading from the initial molecular perturbation by a stressor to an adverse outcome of regulatory relevance (OECD 2017; Villeneuve et al. 2014). AOPs are simplified representations of complex disease processes that identify intermediate events that are essential, biologically relevant, and testable.

This paper will identify and evaluate the evidence and dose–response for key events leading from IR exposure to breast cancer, and describe available assays and opportunities for measuring these events. Hormone exposure during development and into adulthood influences the risk of spontaneous breast cancer and breast cancer following IR, so this paper will review the role of hormones in IR carcinogenesis, and the intersection of hormonal effects with the key events identified here.

## Methods

We used the approach outlined in Kushman and Kraft et al. (Kushman et al. 2013) to construct this AOP. We used review papers (Barcellos-Hoff and Kleinberg 2013; Barcellos-Hoff and Mao 2016; Barcellos-Hoff and Nguyen 2009; Cadet et al. 2017; Committee to Assess Health Risks from Exposure to Low Levels of Ionizing Radiation 2006; Hanahan and Weinberg 2011; Imaoka et al. 2009; Kadhim et al. 2013; Mukherjee et al. 2014; Nguyen et al. 2011a; Ravanat et al. 2014; Ruhm et al. 2016; Smith et al. 2016; Sridharan et al. 2015) to identify the mechanisms by which exposure to ionizing radiation may cause breast cancer. We then used a multipronged approach to identify literature relating to each mechanism. We conducted comprehensive literature searches of PubMed for each mechanism or mechanisms in combination with ionizing radiation focusing on papers since 2006, and used review and primary literature references as well as PubMed and Google Scholar queries to identify additional publications. The resulting publications were manually curated to identify human or animal in vivo or in vitro peer-reviewed primary publications relevant to ionizing radiation and to the events of interest. Papers were additionally evaluated for data quality and clarity before inclusion. Specifically, we looked for studies with three or more samples in each group, and excluded studies with inadequate controls or unclear methods. We ultimately extracted data from over 500 papers. Supplemental Table #1 summarizes the key studies for each topic.

The AOP was constructed following Organization for Economic Co-operation and Development (OECD) guidelines (OECD 2017, 2018b) entered into the OECD AOP Wiki as two separate AOPs for RONS or DNA damage, and submitted to the OECD AOP Development Programme for inclusion in their work plan. Readers may look there for additional details omitted here because of space constraints.

We evaluated the strength of evidence based on the essentiality of each key event to the pathway and to breast cancer using the modified Bradford Hill considerations as outlined in the OECD AOP handbook (Becker et al. 2015; OECD 2017, 2018b). In other words, do studies show that interfering with the key event reduces the occurrence of downstream events and increasing the key event likewise increases downstream events? We highlight the quality and amount of evidence, noting missing or conflicting evidence and identifying factors not addressed in this AOP. We compiled assays available for each of the key events and outcomes and considered their generalizability to mammary tissue.

## The IR and breast cancer AOP

**Fig. 1:**
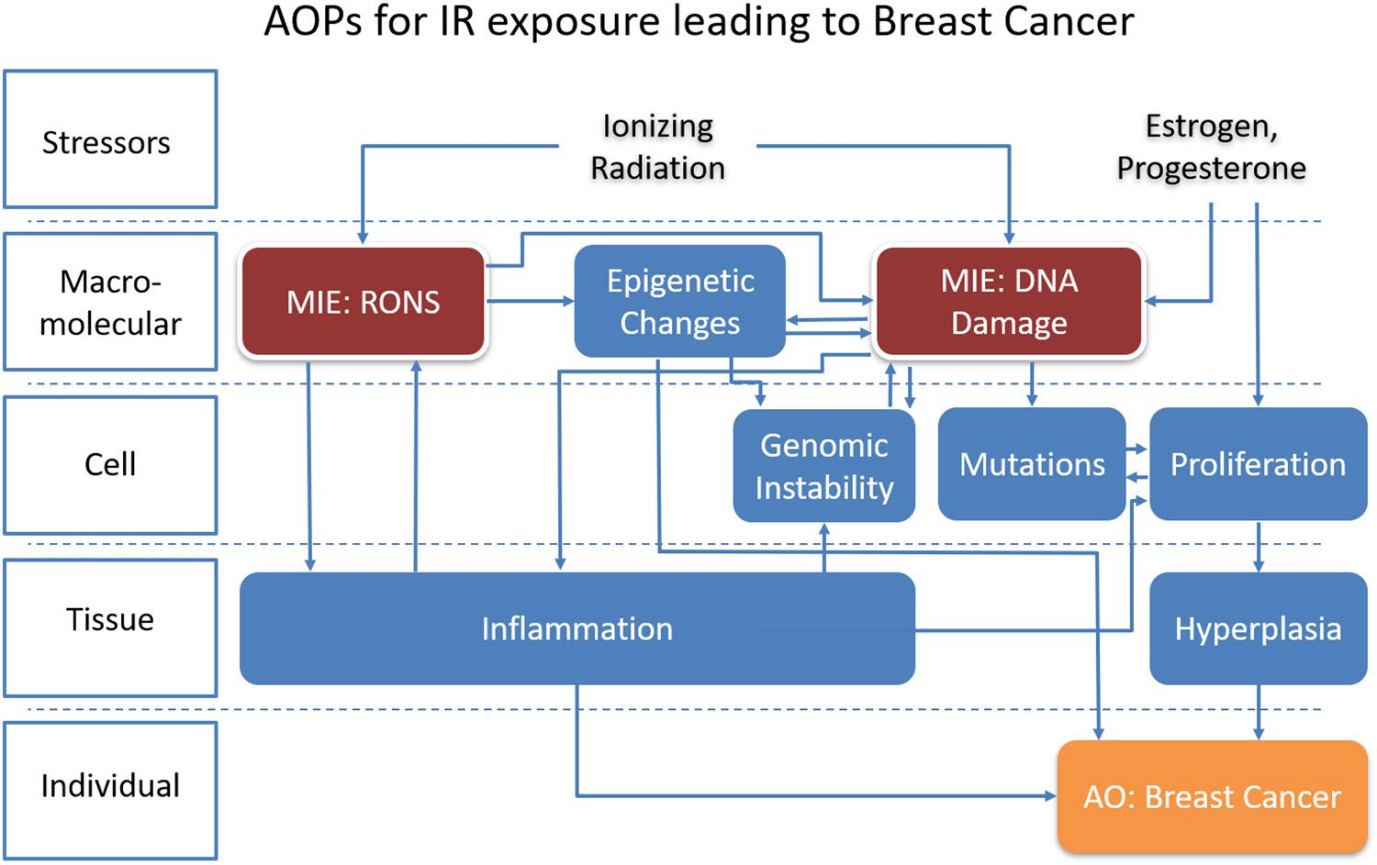
Adverse outcome pathway showing the key events linking the stressor ionizing radiation to breast cancer. Arrows indicate directionality of events: e.g., an increase in the upstream event leads to an increase in the downstream event. The intersection of additional stressors (estrogen and progesterone) with this pathway is included on the right. *MIE* molecular initiating event, the original action caused by the stressor IR in tissue that leads to subsequent events. *AO* adverse outcome. While this pathway is focused on breast cancer as an adverse outcome, DNA damage and GI, mutations, and hyperplasia can be considered adverse outcomes in their own right

The sequence of key events by which ionizing radiation leads to breast cancer is shown in Fig. 1. IR induces two molecular initiating events via oxidation of DNA and proteins: an increase in DNA damage and an increase in reactive oxygen and nitrogen species (RONS). DNA damage leads to increased genomic instability (GI), mutations, proliferation (including clonal selection), hyperplasia, and risk of breast cancer. The increase in RONS also contributes to this pathway via increasing epigenetic changes, GI and DNA damage. Both RONS and DNA damage also initiate multiple inflammatory reactions in the tissue. Inflammation contributes to direct and indirect effects (effects in cells not directly reached by IR) via positive feedback to RONS, DNA damage, and GI and separately increases breast cancer through increased proliferation of cells and tumorigenesis and invasion.

## Ionizing radiation as stressor

Exposure to ionizing radiation (IR) comes from natural and industrial sources such as cosmic rays, radon, or radioactive fuels and wastes and from medical radiation methods such as X-ray imaging, mammography and CT scans, and radiation therapy for cancers. The pattern of energy transferred by IR to matter (linear energy transfer per unit length or LET) (1970) varies between sources. Lower or no LET IR such as mammographic X-rays and some radiation therapies sparsely deposit many individual excitations or small clusters of excitations of low energy (Goodhead 1988) deep into tissue. In contrast, high LET such as alpha particles from radioactive isotopes readily transfer their energy (Goodhead 1994) and, therefore, deposit dense clusters of energy closer to the tissue surface (Goodhead 1988). These different energy deposition patterns contribute to differences in radiation effects including the pattern of DNA damage.

## Breast cancer as adverse outcome (AO)

Exposure to ionizing radiation is a well-established risk factor for many cancers including breast cancer (Ozasa et al. 2012; Preston et al. 2007). Women exposed to the atomic bomb in Japan (exposure is mostly low LET gamma IR with some inhaled high LET alpha and beta particles) (Little and McElvenny 2017), or to therapeutic radiation for benign disorders (Eidemuller et al. 2015), childhood cancer (Henderson et al. 2010; Moskowitz et al. 2014), or contralateral breast cancer (Neta et al. 2012) (often low LET X-rays but also higher LET beta radiation), or to frequent chest X-rays including TB fluoroscopy (Bijwaard et al. 2010; Ma et al. 2008) show a significant increase in breast cancer with radiation exposure. Ionizing radiation also increases mammary tumors in rodents, and sensitivity varies by species and strain (Bijwaard et al. 2010; Ma et al. 2008). See Supplemental Tables 2 and 3 for a summary of key studies examining ionizing radiation and breast and solid cancers in epidemiology and rodent studies, including interactions between IR and hormonal manipulation.

### Why breast?

The key events described here are likely relevant to all tissues after exposure to IR, and are particularly relevant to the breast. While ionizing radiation causes many kinds of cancers including leukemia, lung, bladder, and thyroid cancers (Committee to Assess Health Risks from Exposure to Low Levels of Ionizing Radiation 2006; Preston et al. 2007), breast cancers are among the cancers most increased by exposure to ionizing radiation (Preston et al. 2007).

The lengthy and hormone-dependent developmental trajectory of the breast is likely to be a major factor in its susceptibility to breast cancer in general and to breast cancer from ionizing radiation in particular. Although at first examination, breast cancer from ionizing radiation and hormones involve very different processes, in fact the hormone-dependent and ionizing radiation pathways of carcinogenesis intersect at multiple points along the trajectory of breast development and function, leaving the hormone-exposed breast especially vulnerable to radiation.

One major mechanism promoting breast cancer from ionizing radiation is the proliferation of breast stem cells. Most breast cancers form from epithelial cells, and the growth of these cells in the breast is limited until hormones rise during puberty, when stem cells proliferate to form ductal structures (Hinck and Silberstein 2005; Sternlicht et al. 2005). Stem cells are considered important to initiation because of their long life and capacity to pass on mutations to many progeny (Imaoka et al. 2009; Russo 2015). Breast tissue is responsive to estrogen and progesterone, reproductive hormones that rise at puberty and stimulate cellular proliferation with each reproductive cycle and in pregnancy. These hormonal proliferative cycles increase the risk of cancer in breast tissue (Brisken et al. 2015). IR increases the long-term proliferation of stem cells in pubertal but not adult mammary gland (Datta et al. 2012a; Nguyen et al. 2011b; Snijders et al. 2012; Suman et al. 2012; Tang et al. 2014). Replication of stem cells in the IR-exposed breast is, therefore, particularly elevated during puberty, likely contributing to the increased susceptibility to breast cancer from IR at this age.

Development continues to a lesser degree after puberty with each menstrual cycle until pregnancy, when another major hormone-dependent proliferation and subsequent differentiation and decline of stem cells is coupled with a decline in hormone-sensing epithelial cells (Anderson et al. 2007; Dall et al. 2017; Oakes et al. 2006b). This pregnancy-related decrease in hormone-sensing and stem cells does not occur in first pregnancies at older ages and may explain the lack of protection against spontaneous breast cancer afforded by the first parity in older women (Dall et al. 2017). This theory underlies the current efforts to prevent breast cancers by induction of terminal differentiation (mimicking pregnancy) in teenagers (Santucci-Pereira et al. 2013).

Another special vulnerability of the breast to IR is a byproduct of proliferation:mutations. Replication itself increases the likelihood of mutations, which add to mutations arising from IR and increase the likelihood of oncogenic transformation (Atashgaran et al. 2016). Furthermore, the high replication rate of mammary stem cells during puberty and pregnancy increases reliance on homologous recombination pathways (Kass et al. 2016) which shift to error-prone non-homologous end joining to respond to DNA damage from IR (Chang et al. 2015). Long-term disruption of these homologous repair (HR) processes by polymorphisms in genes like BRCA or by IR-induced mutation can also increase mutation rates and increase tumorigenesis (Mahdi et al. 2018). The consequence of mutations in stem cells is significant, since these cells can clonally expand to generate many mutated progeny. However, errors in stem cell division may not be the sole or primary factor driving cancer from radiation, since excess cancer risk for solid cancers at different sites from the atomic bomb is not clearly related to the number of stem cell divisions at that site (Tomasetti et al. 2017).

The elevated estrogen associated with normal development and the estrous cycle may also have direct effects that further complement the carcinogenic effects of IR. Estrogen directly increases oxidative stress in virgin (but not parous) mice (Yuan et al. 2016), interferes with DNA repair (Li et al. 2014; Pedram et al. 2009) increases DNA damage and mutations (Mailander et al. 2006), and increases TGF-β (Jerry et al. 2010). These effects are seen at physiological concentrations of estrogen, and each creates the potential for additive or synergistic interactions.

Inflammation from the estrous cycle may also contribute to tumorigenesis following IR. Cytokines and macrophages play an integral role in mammary gland development and ductal elaboration, with alternating inflammatory, immune surveillance, and phagocytic activity occurring over each estrous cycle (Atashgaran et al. 2016; Brady et al. 2016; Hodson et al. 2013). This inflammation could theoretically increase IR-induced DNA damage and mutations and promote tumorigenic and invasive characteristics.

The enhancement of IR-induced tumorigenesis by the estrous cycle may be replicated or further enhanced by exogenous endocrine disrupting chemicals. Indeed, evidence suggests that BPA (and presumably other estrogenic chemicals) exposure in utero can increase the mammary gland’s response to progesterone during puberty (Brisken et al. 2015). This enhancement would presumably also increase the risk of breast cancer from ionizing radiation, which rises with estrogen exposure and the number of menstrual cycles (De Bruin et al. 2009; Inskip et al. 2009; Moskowitz et al. 2014; Sigurdson et al. 2009; Travis et al. 2003).

### Breast cancer risk factors

Younger age of exposure, exposure to estrogen and progesterone, pregnancy, and genetic susceptibility influence breast cancer risk from IR in people (Berrington de Gonzalez et al. 2010; Boice et al. 1991; Land et al. 2003; Ma et al. 2008; Miller et al. 1989; Preston et al. 2007; Stovall et al. 2008). In rodents, risk is highest for IR exposures during mammary gland development and puberty (Imaoka et al. 2011, 2013, 2017) and lower for embryonic, adult (Imaoka et al. 2011, 2013), and post-estrous rats (Bartstra et al. 1998).

While IR exposure during breast development increases risk, pregnancy or parity is protective against breast cancer from later radiation in people (Brooks et al. 2012a; Land et al. 1994), similar to spontaneous and chemical carcinogen-induced mammary tumors in rodents (Britt et al. 2007; Dall et al. 2017; Imaoka et al. 2009; Rajkumar et al. 2007; Russo 2015). In rodents, IR exposure during or shortly after pregnancy increases mammary cancer (Holtzman et al. 1982; Inano et al. 1991, 1996b; McDaniel et al. 2006; Suzuki et al. 1994), consistent with increased susceptibility due to development and proliferation in the mammary gland during pregnancy. There is also a “risk bump” of pregnancy-associated breast cancer observed in humans (Lambe et al. 1994).

As with spontaneous breast cancer, the reproductive hormones estrogen and progesterone increase the risk of breast cancer following ionizing radiation. Breast cancer rates following IR are lower in women who undergo premature menopause (De Bruin et al. 2009; Inskip et al. 2009; Moskowitz et al. 2014; Travis et al. 2003). Genetic variation in estrogen signaling also affects risk (Sigurdson et al. 2009), Similarly, exposure to estrogen or the synthetic estrogen diethylstilbestrol (DES) is associated with more tumors (particularly adenocarcinomas) in rats following IR (Broerse et al. 1987; Holtzman et al. 1979, 1981; Inano et al. 1991; Segaloff and Maxfield 1971; Shellabarger et al. 1976; Solleveld et al. 1986) including male (Inano et al. 1996a) and ovariectomized rats (Inano et al. 1995; Yamanouchi et al. 1995). Conversely, ovariectomy (Clifton et al. 1985; Cronkite et al. 1960; Solleveld et al. 1986) and the anti-estrogen tamoxifen (Lemon et al. 1989; Peterson et al. 2005; Welsch et al. 1981) reduce tumors from IR.

Breast cancer risk from IR in postmenopausal atomic bomb survivors increases with and may be partially mediated by increased serum estrogen (Grant et al. 2018), which may in turn be mediated by inflammatory factors (Frasor et al. 2008; Salama et al. 2009). In addition, one study reports that IR can increase circulating estrogen in rodents (Suman et al. 2012). To our knowledge, no publications have attempted to replicate these findings. Progesterone also promotes mammary carcinogenesis after IR in rodents, but its effect depends on the developmental state of the mammary gland: moderate doses promote carcinogenesis in the mature but not immature mammary gland, but high doses can be protective, possibly by differentiating the gland in a manner akin to pregnancy (Inano et al. 1995; Takabatake et al. 2018; Yamanouchi et al. 1995).

IR may increase the risk of ER- breast cancer in particular (Alkner et al. 2015; Castiglioni et al. 2007; Dores et al. 2010; Horst et al. 2014; Huang et al. 2000; Neta et al. 2012; VoPham et al. 2017), although some studies report no difference between risk of ER+ and ER− cancers (Ma et al. 2008; Miura et al. 2008). This effect depends on the presence of estrogen or developmental state: in animals, tumors formed after IR in the absence of estrogen (ovariectomized animals) are often ER−, while those formed in the presence of estrogen or DES are often ER + (Inano et al. 1995) and those formed in the presence of estrogen and progesterone are almost always ER + (Inano et al. 1995; Yamanouchi et al. 1995). Breast tumors can change from ER+ to ER− as the tumor advances or in response to ER antagonists (Lindstrom et al. 2012; Szostakowska et al. 2019).

An early study of women exposed to the atomic bomb suggested that a surge in early onset cancers arose from genetically susceptible populations (Land et al. 1993). Studies of genetic polymorphisms in people and strain variation in rodents support a contribution of genetic background to breast cancer risk from IR (Andrieu et al. 2006; Bernstein et al. 2010; Bernstein et al. 2013; Broeks et al. 2007; Brooks et al. 2012b; Imaoka et al. 2007; Millikan et al. 2005; Pijpe et al. 2012; Rivina et al. 2016; Shellabarger 1972; Sigurdson et al. 2009; Vogel and Turner 1982).

## RONS (MIE)

### Evidence of activation by IR

Reactive oxygen and nitrogen species (RONS) are oxygen- and nitrogen-based molecules that often contain or generate free radicals, including superoxide ([O_2_]•−), hydrogen peroxide (H_2_O_2_), hydroxyl radical ([OH]•), lipid peroxide (ROOH)*,* nitric oxide ([NO]•, and peroxynitrite ([ONOO–]) (Dickinson and Chang 2011; Egea et al. 2017). RONS are generated in the course of cellular respiration, metabolism, cell signaling, and inflammation (Dickinson and Chang 2011; Egea et al. 2017).

We include both reactive oxygen (ROS) and nitrogen (RNS) species in this key event for several reasons. First, reactive species of both types interact and produce overlapping effects (Calcerrada et al. 2011; Mikkelsen and Wardman 2003). Second, because the most common methods used to detect their presence are fairly non-selective (Kalyanaraman et al. 2012; Mikkelsen and Wardman 2003) and thus the majority of evidence for their activation by IR does not distinguish between them. Third, evidence suggests that both ROS and RNS are produced after IR and are involved in the generation of downstream events (addressed below).

**Fig. 2:**
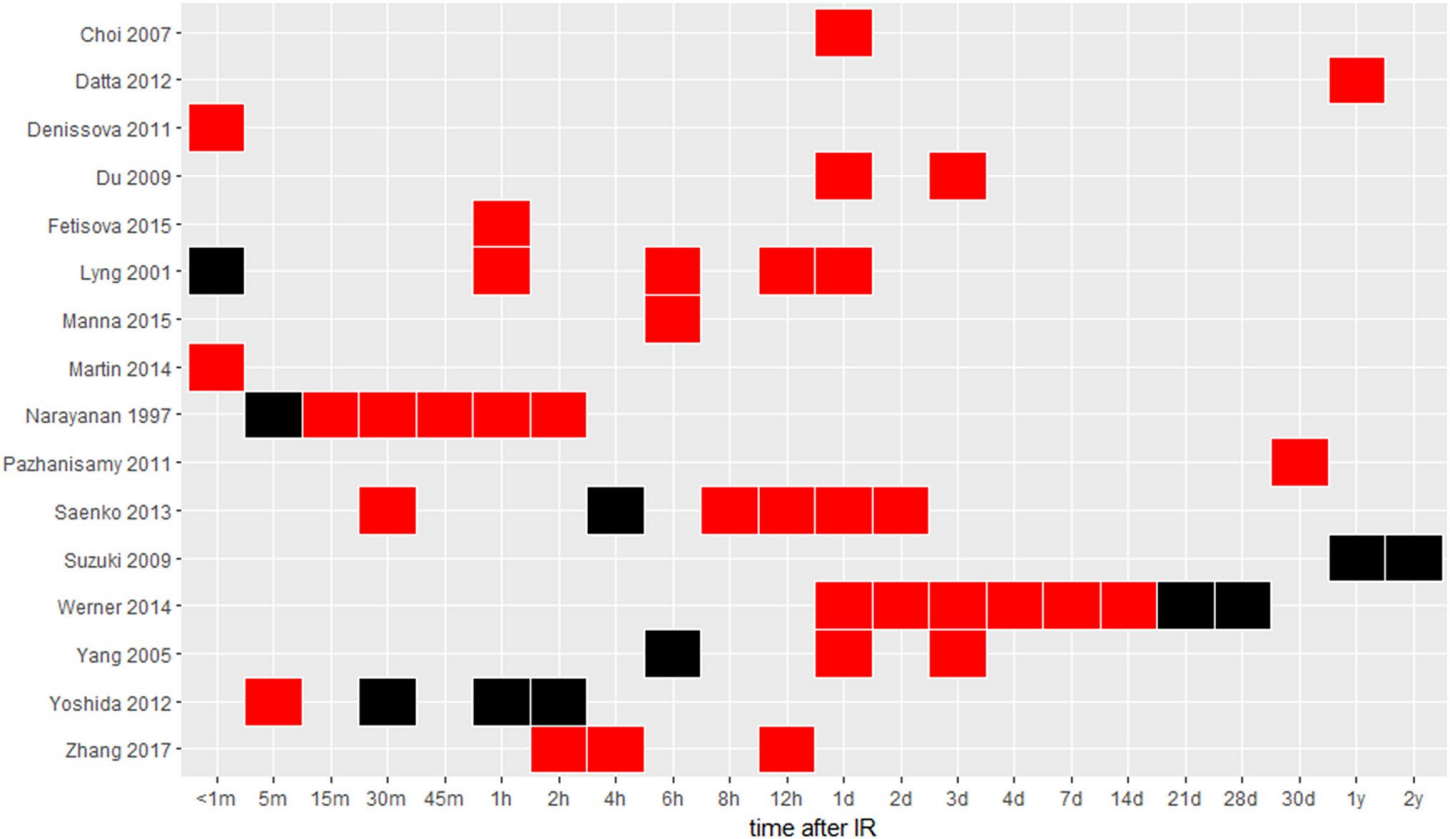
Intracellular RONS at varying times after IR. RONS are measured with DCFH (dichlorodihydrofluorescein diacetate), a common but non-specific fluorescent indicator of intracellular oxidants. Red indicates elevated RONS; black indicates that RONS was measured but not elevated. Each reference is listed on the *y*-axis. Cell types are mostly embryonic or immortalized fibroblasts, keratinocytes, epithelial cells, and immune cells—none are mammary/breast (color figure online)

RONS levels increase at multiple time points in vitro and in vivo following IR indicating a persistent effect (Fig. 2) (Ameziane-El-Hassani et al. 2015; Choi et al. 2007; Das et al. 2014; Datta et al. 2012b; Denissova et al. 2012; Du et al. 2009; Lyng et al. 2001; Manna et al. 2015; Martin et al. 2014; Narayanan et al. 1997; Pazhanisamy et al. 2011; Saenko et al. 2013; Werner et al. 2014; Yang et al. 2005; Yoshida et al. 2012; Zhang et al. 2017). Ionizing radiation initially generates RONS by direct hydrolysis of water molecules. An early (15 min) and later (days to weeks) elevation in RONS is associated with increased NADPH-oxidase production of superoxide and H_2_O_2_ (Ameziane-El-Hassani et al. 2015; Narayanan et al. 1997). Intermediate (hours to days) and chronic RONS elevation has been associated with mitochondrial respiration (Datta et al. 2012b; Dayal et al. 2009; Saenko et al. 2013) driven by nitric oxide signals and cell division (Suzuki et al. 2009). IR-exposed cells show elevated oxidative activity up to a year after IR exposure of the animal (Datta et al. 2012b; Pazhanisamy et al. 2011). For RNS in particular, mitochondrial and NOS1-dependent NOS increase NO and peroxynitrate at 1–30 min after IR, peaking around 5 min (Kostyuk et al. 2012; Leach et al. 2002). Mitochondrial and NF-κB dependent NO concentrations increase again around 8–24 h (Dong et al. 2015; Ha et al. 2010; Saenko et al. 2013; Zhou et al. 2008). RONS (Buonanno et al. 2011; Lyng et al. 2001; Narayanan et al. 1997; Yang et al. 2005) including RNS (Shao et al. 2008; Zhou et al. 2008) is also increased in neighboring or bystander cells—cells not directly exposed to IR—via H_2_O_2_, NO, TNF-α, and TGF-β (Narayanan et al. 1997; Shao et al. 2008; Zhou et al. 2008) as well as COX2 and NF-κB activation and decreased SOD2 (Chai et al. 2013b; Tian et al. 2015; Zhou et al. 2008). Measures of RONS after IR in breast or breast cancer cells are rare, but generally support increased RONS minutes or days after IR (Bensimon et al. 2016; Jones et al. 2007; Leach et al. 2001).

A few studies have measured RONS at multiple or lower doses (< 1 Gy) of ionizing radiation to establish a clear dose–response relationship, and those that have tested multiple doses performed their measures at a range of time points after IR, making it difficult to establish a clear narrative. Immediately after IR, one study at doses above 1 Gy finds that IR increases the number of cells with elevated RONS rather than the degree of RONS in each cell (Leach 2001) and another at doses below 1 Gy reports non-linear dose-dependence of RONS species with a maximum response around 0.4 Gy (Narayanan et al. 1997). RONS increases between 0.5 and 12 Gy IR at 1–2 days (Jones et al. 2007; Saenko et al. 2013) but plateaus at 1 Gy (Werner et al. 2014) at 7 days. The only study explicitly measuring reactive nitrogen species (RNS) at different doses of IR (in endothelial cells) also reports non-linear dose-dependence at 3 h, with a nadir around 0.1 Gy (Kostyuk et al. 2012). RONS increases dose-dependently in extracellular media (Driessens et al. 2009) and in bystander cells not directly exposed to IR (Buonanno et al. 2011; Narayanan et al. 1997).

### Essentiality of RONS

**Essentiality is high.** RONS are sufficient to trigger subsequent key events in this AOP. Extracellularly applied or intracellularly generated ROS (which also facilitates the formation of RNS) are capable of creating DNA damage in vitro including base damage, single- and double-strand breaks, and chromosomal damage (Berdelle et al. 2011; Dahm-Daphi et al. 2000; Driessens et al. 2009; Gradzka and Iwanenko 2005; Ismail et al. 2005; Lorat et al. 2015; Nakamura et al. 2003; Oya et al. 1986; Stanicka et al. 2015) and mutations (Ameziane-El-Hassani et al. 2010; Sandhu and Birnboim 1997; Seager et al. 2012; Sharma et al. 2016). Similarly, decreased antioxidant activity and higher RONS are observed in cells with GI (Bensimon et al. 2016; Buonanno et al. 2011; Datta et al. 2012b; Dayal et al. 2008; Kim et al. 2006; Limoli and Giedzinski 2003; Limoli et al. 2003; Natarajan et al. 2007; Owens et al. 2012; Pazhanisamy et al. 2011; Thomas et al. 2012). RONS are also involved in the activation of inflammation (Nakao et al. 2008; Zhang et al. 2017) and epigenetic effects (Bernal et al. 2013; Galligan et al. 2014; Kloypan et al. 2015; Simone et al. 2009; Yang et al. 2014).

The strongest evidence for the essentiality of RONS in breast cancer from IR comes from studies, showing that reducing RONS with antioxidants including catalase or SOD or NOX/DUOX inhibitors also reduces downstream key events including DNA damage and GI in multiple types of irradiated cells and bystander cells in vivo and in vitro after IR (Ameziane-El-Hassani et al. 2010, 2015; Azzam et al. 2002; Bensimon et al. 2016; Choi et al. 2007; Dayal et al. 2008, 2009; Douki et al. 2006; Jones et al. 2007; Manna et al. 2015; Ozyurt et al. 2014; Pazhanisamy et al. 2011; Tartier et al. 2007; Tian et al. 2015; Weyemi et al. 2015; Winyard et al. 1992; Yang et al. 2007b, 2005), while inhibiting NO in particular inhibits DNA damage and mutations in bystander cells (Han et al. 2010; Tartier et al. 2007; Yang et al. 2007b; Zhou et al. 2008). Antioxidant activity also reduces the inflammatory response to IR in animals and cultured skin cells (Berruyer et al. 2004; Das et al. 2014; Ezz et al. 2018; Haddadi et al. 2017; Khan et al. 2015; Ozyurt et al. 2014; Sinha et al. 2011, 2012; Zetner et al. 2016; Zhang et al. 2017) and the epigenetic effects of IR in vitro (Bernal et al. 2013; Kloypan et al. 2015; Simone et al. 2009). RONS also drives proliferation directly (Han et al. 2010) and via cell death and compensatory proliferation following damage to DNA and important cellular macromolecules (Nishida et al. 2013).

None of the studies addressing RONS and inflammation are in mammary gland/breast, but a limited number of studies in mammary and breast cells do support the essentiality of RONS to DNA damage or cancer (Bensimon et al. 2016; Dutta et al. 2014) including after IR (Inano et al. 1999; Jones et al. 2007).

RONS act in multiple ways to promote downstream key events, not only directly damaging DNA and proteins but also modifying signal transduction pathways and epigenetic mechanisms (Gao and Schottker 2017; Mikkelsen and Wardman 2003). Examples include inflammatory pathways like TNF-α and IL-6, transcription factors like NF-κB, and methylation of histones (Eskiocak et al. 2010; Galligan et al. 2014; Kloypan et al. 2015; Yang et al. 2014; Zhou et al. 2008).

### Data gaps and uncertainties

Although many studies support the link between IR, RONS, and DNA damage, a few RONS studies use mammary tissue, multiple doses, doses below 1 Gy, or explicitly measure RNS presenting an addressable data gap.

Despite the effectiveness of antioxidants in decreasing all forms of DNA damage, externally applied RONS is less effective than IR at generating double-strand breaks and mutations (Dahm-Daphi et al. 2000; Gradzka and Iwanenko 2005; Ismail et al. 2005; Sandhu and Birnboim 1997). One possible explanation for this discrepancy is that IR may elicit a higher concentration of localized RONS than can be achieved with external application of H_2_O_2_. IR deposits energy and oxidizes molecules within a relatively small area over a rapid timescale potentially permitting a very high local concentration which could precede or overwhelm local buffering capacity. In contrast, extracellularly applied H_2_O_2_ would interact with many antioxidants and other molecules on its way to the nucleus, where the concentration would slowly reach a lower steady state.

To our knowledge, no experiments have tested whether elevating intracellular RONS alone in one group of cells can cause bystander effects in another.

## DNA damage, GI, and mutation (MIE & KEs)

### Evidence of activation by IR and RONS

IR and RONS cause immediate and delayed DNA damage and GI. Both of these forms of DNA damage contribute to changes in DNA sequence, i.e., mutations. DNA changes include to the base of the nucleotide (with guanine particularly vulnerable because of its low redox potential), a break in the sugar (deoxyribose)-phosphate backbone creating a single-strand break (David et al. 2007), or simultaneous proximal breaks in both strands of DNA to form double-strand breaks (Ameziane-El-Hassani et al. 2015; Berdelle et al. 2011; Dahm-Daphi et al. 2000; Driessens et al. 2009; Haegele et al. 1998; Han et al. 2010; Lorat et al. 2015; Nakamura et al. 2003; Oya et al. 1986; Pouget et al. 2002; Roots et al. 1990; Rothkamm and Lobrich 2003; Sandhu and Birnboim 1997; Seager et al. 2012; Sharma et al. 2016; Stanicka et al. 2015; Ward 1988; Werner et al. 2014; Yang et al. 2013). Double-strand breaks are considered more destructive and mutagenic than lesions or single-strand breaks, and higher densities of double-strand breaks generate chromosomal abnormalities including changes in chromosomal number, breaks and gaps, translocations, inversions, and deletions (Behjati et al. 2016; Haag et al. 1996; Morishita et al. 2016; Ponnaiya et al. 1997b; Unger et al. 2010; Yang et al. 1992, 1997).

The type and amount of DNA damage depends on both the quality and dose of radiation. The pattern of energy transferred by IR to matter (linear energy transfer per unit length or LET) (1970) varies between sources. Lower or no LET IR such as mammographic X-rays and some radiation therapies sparsely deposit many individual excitations or small clusters of excitations of low energy (Goodhead 1988) deep into tissue. In contrast, high LET such as alpha particles from radon readily transfer their energy (Goodhead 1994) and, therefore, deposit dense clusters of energy closer to the tissue surface (Goodhead 1988).

These different energy deposition patterns contribute to differences in radiation effects including the pattern of DNA damage. Higher LET radiation such as alpha particles generates more complex clusters of damage including more frequent double-strand breaks (Nikitaki et al. 2016; Ottolenghi et al. 1997; Rydberg et al. 2002; Watanabe et al. 2015) and other chromosomal abnormalities (Anderson et al. 2002; Yang et al. 1997), while lower LET radiation (gamma rays, X-rays) and RONS generate more oxidized base damage and single-strand breaks (Douki et al. 2006). Although complex damage is more likely to occur from high LET IR, low LET IR and RONS can also generate complex damage, even from a single particle or photon (Ravanat et al. 2014; Sharma et al. 2016; Sutherland et al. 2002). Complex damage is more difficult to repair, making it more detrimental to the cell and the organism than a single point of damage (Kuhne et al. 2000; Pinto et al. 2005; Rydberg et al. 2005; Stenerlow et al. 2000).

DNA damage is also observed in in cells not directly in the path of ionizing radiation and at a delay following exposure. These indirect or bystander effects are mediated by multiple factors including RONS (Yang et al. 2005), TGF-β (Dickey et al. 2009), and other cytokines (Havaki et al. 2015).

Most evidence suggests that nucleotide damage, single-strand breaks, clustered damage, and double-strand breaks increase linearly with IR dose over a wide range (0.001–80 Gy) (Asaithamby and Chen 2009; Beels et al. 2010; Ojima et al. 2008; Roots et al. 1990; Rothkamm and Lobrich 2003; Rydberg et al. 2002; Sutherland et al. 2002; Yang et al. 2005). The low LET dose–response of chromosomal aberrations and other mis-rejoining of DNA is linear or supralinear (Iwasaki et al. 2011; Ryu et al. 2016; Tanaka et al. 2009; Yang et al. 1997) at lower doses (0.01 Gy) (Iwasaki et al. 2011; Schiestl et al. 1994) and dose–response upwardly curving at high-dose rates (0.89 Gy/day) and higher doses (10–80 Gy) (Olipitz et al. 2012; Rydberg et al. 2005; Tanaka et al. 2009), while the response to high LET radiation is linear (Jones et al. 2007; Yang et al. 1997). DNA damage measured in bystander cells 1 h to 3 days after exposure is also dose-dependent at very low doses, but may approach a maximum around 0.1 Gy (Ojima et al. 2008; Yang et al. 2007b).

GI is the recurrent appearance of DNA and chromosomal damage and mutations. DNA damage following ionizing radiation in directly and indirectly damaged cells is repaired over the first few hours or days (Nikitaki et al. 2016), but GI can appear weeks, months, or even years after the initial exposure and persist in subsequent generations of cells in vivo (Datta et al. 2012b; Mukherjee et al. 2012; Pazhanisamy et al. 2011; Snijders et al. 2012) and in vitro (Bensimon et al. 2016; Buonanno et al. 2011; Moore et al. 2005; Natarajan et al. 2007). DNA damage response and dysfunctional telomeres are implicated in the formation of GI (Sishc et al. 2015; Williams et al. 2009; Yu et al. 2001), but the delayed appearance and high frequency of GI and its transmission through media to bystander cells strongly suggest the involvement of non-mutational (epigenetic) mechanisms (Al-Mayah et al. 2012; Rugo et al. 2011; Ullrich and Davis 1999). RONS is also involved in the production of GI (Bensimon et al. 2016; Buonanno et al. 2011; Datta et al. 2012b; Dayal et al. 2008; Kim et al. 2006; Limoli and Giedzinski 2003; Limoli et al. 2003; Natarajan et al. 2007; Owens et al. 2012; Pazhanisamy et al. 2011; Thomas et al. 2012).

Mutations occur when DNA damage is converted to permanent changes in nucleotide sequence. Nucleotide lesions contribute to point mutations when a different nucleotide is mistakenly inserted in place of a damaged nucleotide, sometimes altering the resulting amino acid. Nucleotide lesions also contribute to single-strand breaks, and both lesions and single-strand breaks contribute to double-strand breaks if not repaired before replication. Double-strand breaks contribute to deletions, inversions, translocations, and duplications, and can lead to major chromosomal damage. Complex DNA damage (multiple damaged sites in close proximity) is more difficult and time-consuming to repair, so complex damage is more likely to persist until replication and be converted into mutations.

Because DNA damage and mutation increase the risk of all types of cancers, these events are the subject of established guideline assays (Table 1) and are considered adverse outcomes in their own right.

**Table 1:** Guideline assays and other methods measuring key events in the pathway to breast cancer

Event	Guideline test	Methods	Tissue
Breast cancer	Yes	OECD Test No. 451 and 453, 2-year bioassay for carcinogenicity and combined toxicity and carcinogenicity (OECD 2009a, b)	Mammary
		US National Toxicology Program (NTP), FDA, EPA guidelines for 2-year cancer bioassay and risk assessments (EPA 2005; FDA 2007; NTP 2011b)	Mammary
RONS	No	Fluorescent protein-based probes (Dickinson and Chang 2011; Griendling et al. 2016; Kalyanaraman et al. 2012; Wang et al. 2013)	Any*
		Boronate-based small molecule probes (Dickinson and Chang 2011; Griendling et al. 2016; Kalyanaraman et al. 2012; Wang et al. 2013)	Any*
		EPA ToxCast assay for mitochondrial membrane potential and intracellular superoxide (DHE) (Giuliano et al. 2010)	Primary rat hepatocyte*
DNA damage	Yes	OECD DNA synthesis Test No. 486 for nucleotide excision repair (OECD 1997b)	Mammalian liver cells—only informs primary damage to liver (EFSA Scientific Committee et al. 2017)
		OECD Alkaline comet assay Test No. 489 for single- and double-strand breaks and nucleotide damage (OECD 2016f)	Any*,**
		OECD Chromosomal damage and micronuclei Test No. 473, 475, 483, and 487 (OECD 2016a; OECD 2016b; OECD 2016d; OECD 2016e)	473, 487: Stable cell line, esp lymphocyte (no validated mammary)*
			475: Mammalian bone marrow*,**
			483: Rodent sperm cells*,**
	No	Electrophoretic methods for finding strand breaks or specific DNA lesions: high-throughput comet assay (Ge et al. 2014; Sykora et al. 2018), pulsed field gel electrophoresis (PFGE) (Nikitaki et al. 2015; Sutherland et al. 2003)	Any*
		Direct measurement of DNA lesions via HPLC–MS/MS (Ravanat 2012)	Any**
		Immunostaining using antibodies to label DNA damage repair proteins (H2AX, XRCC2, OGG1, etc.) coupled with microscopy or flow cytometry (Lobrich et al. 2005; Nikitaki et al. 2015, 2016; Ojima et al. 2008; Rothkamm and Lobrich 2003). Can detect clustered lesions (Nikitaki et al. 2016)	Any*,**
		EPA ToxCast anti-p53 assay (Giuliano et al. 2010)	HEPG2 cells*
Mutation	Yes	OECD Ames Test No. 471 (OECD 1997a)	Bacteria*
		OECD Hprt or Xprt Test No. 476 (OECD 2016c)	Selected lines, not mammary*
		OECD Transgenic Rodent Somatic and Germ Cell Gene Mutation Assays Test No. 488 (OECD 2013)	Any (performed in mammary gland (Jakubczak et al. 1996)**
		OECD Thymidine kinase Test No. 490 (OECD 2016g)	Lymphoma or lymphoblastoid cell lines*
	No	Transgenic rodent in vivo/in vitro: RaDR-GFP for errors in homologous recombination (sister chromatid exchange) (Sukup-Jackson et al. 2014)	Any (performed in mammary gland (Sukup-Jackson et al. 2014))**
		Transgenic rodent in vitro: many tests, some metabolically characterized (White et al. 2019)	Any (some in mammary) (White et al. 2019)
		Array CGH detects copy number variations in tumors or clonal cells (Bonnet et al. 2012; Gaudet et al. 2003)	Any*
Genomic Instability	No	Many of the above methods for detecting DNA and chromosomal damage and mutation are applied at a range of time points to detect GI (Datta et al. 2012b; Gaudet et al. 2003; Lee et al. 2015; Owens et al. 2012; Rugo et al. 2011; Sishc et al. 2015; Snijders et al. 2012). Multiple time points should be used to find the rate of ongoing damage, and multiple methods used to capture the range of possible GI outcomes (Limoli et al. 1997)	Any*,**
Proliferation, Hyperplasia	No	Change in cell numbers (DNA synthesis markers (in vivo or in vitro), impedance, time lapse imaging, colony formation assay (in vitro)) (Menyhart et al. 2016; Morgan et al. 2020)	Any*
		Immunohistochemistry (Ki67 labeling) (Romar et al. 2016)	Any*,**
		Histology (proliferation, hyperplasia) in in vivo toxicity studies such as 90-day subchronic, 28 day, or 14-day can include longitudinal sectioning of male and female mammary glands and BrDU injection and staining to better detect proliferation (Collins 2018; Makris 2011; Rudel et al. 2011)	Any*,**
		EPA ToxCast BRDU assay (Giuliano et al. 2010)	HepG2 carcinoma line*
Inflammation	No	Detection of inflammatory proteins like IL6, TNF-α: ELISA (El-Saghire et al. 2013; Partridge et al. 2010; Siva et al. 2014), Western Blot (Chai et al. 2013b; Ha et al. 2010), electrophoretic mobility shift assay (Haase et al. 2003), immunostaining (Chai et al. 2013b; Wang et al. 2015), PCR based changes in expression of inflammation-related signals (Azimzadeh et al. 2011; Bouchet et al. 2013; Moravan et al. 2011; Snijders et al. 2012; Wang et al. 2014b)	Any (ideally both target tissue and leukocytes)*,**
		Histology (leukocyte infiltration) (Ebrahimian et al. 2018; Monceau et al. 2013; Moravan et al. 2011)	Any*,**
		EPA ToxCast BioMap assays (Houck et al. 2009)	Various, not including mammary*
Epigenetic changes	No	Global methylation: [^3^H]dCTP extension assay (Koturbash et al. 2016), HPLC for 5mdC (Wang et al. 2014a), quantitative immunoassay for 5-mc (Nzabarushimana et al. 2014)	Any*,**
		Gene-specific methylation: MeDIP-on-chip (Hsu et al. 2015), methylation-sensitive/dependent restriction enzymes and qPCR (Nzabarushimana et al. 2014; Oakes et al. 2006a), bisulfite sequencing (Wang et al. 2014a)	Any*,**
		miRNA expression (Mestdagh et al. 2014): qRT-PCR (Stankevicins et al. 2013; Szatmari et al. 2017), hybridization (e.g., microarray, etc.) (Aypar et al. 2011; Jacob et al. 2013), sequencing (Mestdagh et al. 2014)	Any*,**
		Histone methylation: ChIP (Prior et al. 2016) and immunohistochemistry (Kutanzi and Kovalchuk 2013)	Any*,**
		Gene expression: western blot (Wang et al. 2014a), qRT-PCR (Prior et al. 2016), whole transcriptomics e.g. RNA microarray, RNA-seq (Hrdlickova et al. 2017; Manzoni et al. 2018)	Any*,**

*This test/measurement is not typically conducted in mammary tissue, so validation that results are generalizable is needed

**Verify that test substance reaches the mammary gland

### Essentiality of DNA damage, GI, and mutation

**Essentiality is high.** Although not commonly measured in the same experiments, IR-induced DNA damage, GI, and mutation (Denissova et al. 2012; Fibach and Rachmilewitz 2015; Jones et al. 2007; Padula et al. 2016; Sandhu and Birnboim 1997; Ullrich and Davis 1999; Wazer et al. 1994) precede cell transformation (Ullrich et al. 1996; Unger et al. 2010; Wang et al. 2011; Wazer et al. 1994; Yang et al. 1992, 1997), and tumorigenesis (Adams et al. 1987; Andrieu et al. 2006; Bernstein et al. 2010; Bernstein et al. 2013; Broeks et al. 2007; Brooks et al. 2012b; de Ostrovich et al. 2008; Francis et al. 2009; Francis et al. 2011; Gustin et al. 2009; Little 2009; Millikan et al. 2005; Pijpe et al. 2012; Podsypanina et al. 2008; Poirier and Beland 1994; Radice et al. 1997; Tao et al. 2017; Umesako et al. 2005). Further support for including DNA damage, GI, and mutation in the mechanistic pathway linking ionizing radiation with breast cancer comes from the observation that variants in DNA repair genes increase the risk of mammary tumors in animals after IR (Umesako et al. 2005; Yu et al. 2001) and increase breast cancer after IR (Andrieu et al. 2006; Bernstein et al. 2010, 2013; Broeks et al. 2007; Brooks et al. 2012b; Millikan et al. 2005; Pijpe et al. 2012). BRCA is perhaps the best known DNA repair gene linked with breast cancer risk, and several of these studies have suggested a link between BRCA mutation status and increased susceptibility to breast cancer following ionizing radiation, particularly in women exposed at younger ages (Pijpe et al. 2012).

Outside of the IR literature, multiple studies confirm that mutations can increase proliferation, hyperplasia, and tumors (Podsypanina et al. 2008). Mutations in cell cycle checkpoint, growth factor signaling, and other genes enable mammary epithelial and other cells to proliferate in vitro (Gustin et al. 2009; Higashiguchi et al. 2016; Kouros-Mehr et al. 2006; Shahi et al. 2017; Tao et al. 2011), promote mammary hyperplasia in vivo (Francis et al. 2009), and contribute to tumors when combined with the other mutations in the same pathway (Francis et al. 2011). Restoring function in mutated genes regresses tumors in animals (Martins et al. 2006; Podsypanina et al. 2008). Mutations are common in tumors (CGAN (Cancer Genome Atlas Network) 2012; Garraway and Lander 2013; Greenman et al. 2007; Haag et al. 1996; Stratton et al. 2009; Vandin et al. 2012; Vogelstein et al. 2013; Yang et al. 2015) and tumors are largely clonal, suggesting that individual mutations offer the tumor evolutionary advantages (Begg et al. 2016; Wang et al. 2014c; Yates et al. 2015). GI increases the frequency and duration of DNA damage and mutation after IR, thereby increasing the likelihood of transformation and tumorigenesis (Selvanayagam et al. 1995; Ullrich and Ponnaiya 1998).

The evidence linking DNA damage with epigenetic changes is indirect. The two key events are often observed at the same time or DNA damage precedes epigenetic changes (Koturbash et al. 2016, 2008, 2005, 2006b; Nishida et al. 2013; Pogribny et al. 2005, 2004; Szatmari et al. 2017). However, DNA damage does not always correspond with or precede epigenetic changes (Koturbash et al. 2016, 2006b; Pogribny et al. 2005).

### Data gaps and uncertainties

The majority of research on the effects of IR on DNA damage has been performed in tissues other than mammary gland, but several studies suggest that effects in the mammary gland (and its consequences) would be consistent with the other tissues (Haegele et al. 1998; Hernandez et al. 2013; Jones et al. 2007; Snijders et al. 2012; Soler et al. 2009; Wang et al. 2015) and many of the studies in support of the proliferative and tumorigenic role of mutations are in mammary gland or breast cancers (Behjati et al. 2016; CGAN (Cancer Genome Atlas Network) 2012; de Ostrovich et al. 2008; Francis et al. 2009; Francis et al. 2011; Gustin et al. 2009; Haag et al. 1996; Korkola and Archer 1999; Marusyk et al. 2014; Miura et al. 2008; Morganella et al. 2016; Nik-Zainal et al. 2016; Podsypanina et al. 2008; Radice et al. 1997; Tao et al. 2017; Umesako et al. 2005; Wazer et al. 1994; Yang et al. 2015). Recent studies in other tissues have shown a high frequency of cancer-driving mutations in normal tissues, raising questions about factors leading to clonal selection (Martincorena 2019); and further description of these processes in breast represents a data gap.

All cancers have DNA mutations, but mutation is not always the initiating event. Instead mutation can result from other stressors like proliferation or inflammation. Mammary tumor incidence following ionizing radiation treatment of the animal (as discussed above) or transplanted cells (Maffini et al. 2005; McDaniel et al. 2006) varies significantly by sex and depends on the presence of ovarian hormones. Stroma treated with carcinogens or IR supports the growth of tumors from pre-malignant epithelial cells (although these may have pre-existing mutations) (Barcellos-Hoff and Ravani 2000; Maffini et al. 2004; Nguyen et al. 2011b). In these cases, the hormones or inflammation in the mammary gland may modify the stromal environment to increase the likelihood of cellular proliferation, which amplifies the existing mutated cells and generates new mutations.

Some evidence points to a protective effect of lower dose IR on chromosomal damage, mutation, and transformation in vitro (Azzam et al. 2016; Elmore et al. 2008; Ina et al. 2005; Kakinuma et al. 2012; Sakai et al. 2006; Sasaki et al. 2002; Shin et al. 2010; Wolff et al. 1988; Yamauchi et al. 2008) and chromosomal damage, mutation and thymic lymphomas, and skin cancer in vivo (Ina et al. 2005; Kakinuma et al. 2012; Sakai et al. 2006; Shin et al. 2010; Yamauchi et al. 2008). However, the dose range at which protection is observed varies greatly between target tissue, animal models, and other experimental conditions, suggesting that the protection is not a fixed or immutable response (Tang and Loke 2015). Most animal studies (of chemical genotoxicants) are not sufficiently designed or powered to detect low-dose changes in dose–response and are insufficient to reject the linear no threshold model (Guerard et al. 2015), but a large IR animal cancer study and a systematic meta-analysis of IR protection in animals found no convincing evidence of protection against carcinogenesis (Crump et al. 2012; Tanaka et al. 2007). In people, a recent systematic review and meta-analysis of low-dose IR epidemiology concludes that the evidence does not support protection against total solid cancers (Shore et al. 2017), and although the dose–response for epidemiology of leukemia and lymphoma leave open the possibility of a protective response at low doses, a dose–response model without an inflection or protective effect of IR is recommended (Sasaki et al. 2014). No evidence suggests a protective effect of ionizing radiation on breast cancer, and dose–response relationships are further discussed in Sect. 13.2 below.

## Proliferation and hyperplasia (KEs)

### Evidence of proliferation and hyperplasia following IR

While higher doses of ionizing radiation cause cell death in the short term (especially of dividing cells), IR is associated with proliferation in vitro and in vivo. In vitro, IR promotes proliferation/expansion of p16-suppressed and immortal epithelial populations as well as in bystander CHO cells co-cultured with IR-exposed cells (Han et al. 2010; Mukhopadhyay et al. 2010; Tang et al. 2014). In vivo, IR increases compensatory proliferation in adult rats (Loree et al. 2006), and long-term expression of proliferation in adolescent but not adult mammary gland (Datta et al. 2012a; Snijders et al. 2012; Suman et al. 2012), possibly via the expansion of a population of stem-like cells in vivo (Nguyen et al. 2011b; Tang et al. 2014). This proliferation appears to be associated with TGF-β/Notch activity (Tang et al. 2014) and nitric oxide (Han et al. 2010). Proliferative nodules and hyperplasia appear in mammary terminal-end bud, alveolae, and ducts of rats and mice after exposure to ionizing radiation (Ethier and Ullrich 1982; Faulkin et al. 1967; Imaoka et al. 2006) as well as chemical carcinogens (Beuving et al. 1967a, 1967b; Purnell 1980; Russo et al. 1977).

### Essentiality of proliferation

**Essentiality is high.** Cellular proliferation is a key characteristic of cancer cells (Hanahan and Weinberg 2011). Hyperplasia signals the presence of excess proliferation and represents an intermediate phase in the development of tumorigenesis. Hyperplasia is generally considered to be an adverse outcome in its own right.

Proliferation occurs following the release of inhibitory controls limiting entry into the cell cycle, and oncogenic mutations act via these same pathways to generate abnormal proliferation (Hanahan and Weinberg 2011; Weber et al. 2017), including in mammary gland (Francis et al. 2009, 2011; Garbe et al. 2009; Gustin et al. 2009; Weber et al. 2017). Inhibitory signals such as contact inhibition or TGF-β (Francis et al. 2009; Polyak et al. 1994) stabilize the mechanisms limiting entry into the cell cycle. Proliferative signals such as those following progesterone or estrogen (Croce 2008; Weber et al. 2017) or compensatory proliferation after apoptosis (Fogarty and Bergmann 2017) relieve inhibition and enable cells to enter the cell cycle. Mutations that inactivate inhibitory signals (tumor suppressors) or activate proliferative signals (oncogenes) promote proliferation outside of the normal biological context (Francis et al. 2011; Gustin et al. 2009; Hanahan and Weinberg 2011; Weber et al. 2017). Abnormal proliferation is typically met with apoptosis or senescence, so additional mutations or other mechanisms are required to escape these additional levels of control for proliferation to continue indefinitely (Fernald and Kurokawa 2013; Garbe et al. 2009; Shay and Wright 2011).

Proliferation is required for DNA damage and replication errors to be integrated into the genome as mutations (Kiraly et al. 2015). Proliferation can also promote the expansion and clonal selection of existing cells with proliferative mutations. Genomic mutations favoring further proliferation are positively selected from among the expanded cells, resulting in the accumulation of mutational errors and moving the organism further towards cancer. Different clonal populations can also collaborate to promote growth as observed in mammary gland (Franco et al. 2016; Marusyk et al. 2014).

Increasing proliferation leads to hyperplasia in mammary gland (Korkaya et al. 2009). During mammary tumorigenesis following IR in rodents, proliferating foci precede the development of tumors (Haslam and Bern 1977; Purnell 1980) and form tumors more effectively than non-proliferating tissue (Beuving 1968; Deome et al. 1959; Rivera et al. 1981). Adenocarcinomas in mammary gland appear to preferentially form from terminal-end bud hyperplasia (Haslam and Bern 1977; Purnell 1980; Russo et al. 1977) in rats, similar to the origin of many breast cancers for humans and for some mice after IR (Medina and Thompson 2000).

Supporting the essentiality of these proliferative processes to tumorigenesis, ACI rats that exhibit no mammary proliferation or hyperplasia following IR are resistant to tumors following IR (Kutanzi et al. 2010). Interventions reducing proliferation in susceptible PyVT and BALB/c mice also reduce mammary tumors (Connelly et al. 2011; Luo et al. 2009; Tang et al. 2014).

### Data gaps and uncertainties

Some studies report carcinogenesis in the absence of hyperplasia (Sinha and Dao 1974) and others do not find increased tumorigenesis from transplanted hyperplasia (Beuving et al. 1967a; Haslam and Bern 1977; Sinha and Dao 1977).

## Inflammation (KEs)

### Evidence of activation by IR

Inflammatory pathways are commonly activated in mammary gland (Barcellos-Hoff et al. 1994; Bouchard et al. 2013; Datta et al. 2012a; Dickey et al. 2009; Snijders et al. 2012; Wang et al. 2015) and in breast and mammary cancers following IR (Illa-Bochaca et al. 2014; Nguyen et al. 2013, 2011b).

### Essentiality of inflammation

**Essentiality is moderate.** While inflammation in response to tissue damage or infection is typically resolved without lasting effects, excess initial inflammation, chronic inflammation, and anti-inflammatory signaling (via fibrosis) can contribute to chronic inflammatory diseases including cancer (Perez et al. 2014). Such dysregulation of inflammation may be related to continual competition of inflammatory and anti-inflammatory factors, which can lead to chronic wounds (Eming et al. 2014).

Tumors and tumor cells exhibit features of inflammation, and inflammation is generally understood to promote transformation and tumor progression by supporting multiple hallmarks of cancer including oxidative activity and DNA damage, survival and proliferation, angiogenesis, and invasion and metastasis (Esquivel-Velazquez et al. 2015; Hanahan and Weinberg 2011; Iliopoulos et al. 2009).

Many of these cancer promoting effects of inflammation can be seen following exposure to ionizing radiation (Bisht et al. 2003; Bouchard et al. 2013; Elahi et al. 2009; Illa-Bochaca et al. 2014; Nguyen et al. 2011b, 2013). Polymorphisms in inflammation genes are associated with breast cancer risk from IR in radiation technologists (Schonfeld et al. 2010) and with susceptibility to intestinal adenoma following IR in mice (Elahi et al. 2009). Cytokines TGF-β and IL6 transform primary human mammospheres and pre-malignant mammary epithelial cell lines in vitro and make them tumorigenic in vivo (Iliopoulos et al. 2009; Nguyen et al. 2011b; Sansone et al. 2007), and inflammation-related factors COX2 and TGF-β are required for the full effect of IR on DNA damage and transformation in vitro and mammary tumor growth and invasion in vivo (Bisht et al. 2003; Nguyen et al. 2011b). Further evidence for inflammation comes from tumors arising from mammary epithelial cells transplanted into IR-exposed cleared fat pads: inflammation-related genes and pathways are upregulated or enriched in the gene expression patterns of these indirectly IR-induced tumors (Illa-Bochaca et al. 2014; Nguyen et al. 2013, 2011b).

Activation of an inflammatory NF-kB/IL6/STAT3 signaling pathway generates cancer stem cells in breast cancer cells in vitro (Iliopoulos et al. 2009, 2010, 2011) and is also implicated in colon and other cancers (Iliopoulos et al. 2010). The inflammation-related transcription factor NF-kB also contributes to mammary tumorigenesis and metastasis in PyVt mice, in which mammary tumors are induced by expression of an MMTV-driven oncogene (Connelly et al. 2011).

One mechanism of cancer promotion involves oxidative activity and DNA damage: inflammation in response to IR increases oxidative activity in a positive feedback loop leading to increased DNA lesions, GI, and mutations. Oxidative activity mediates the increase in inflammatory (TNF-α and neutrophil) markers (Ozyurt et al. 2014), and oxidative activity is increased by direct treatment with TNF-α or neutrophils (Jackson et al. 1989; Natarajan et al. 2007; Yan et al. 2006; Yang et al. 2007a) or after IR mediated by Il13, TNF-α, CCL2, COX2, and TLR9 (Ameziane-El-Hassani et al. 2015; Jackson et al. 1989; Kostyuk et al. 2012; Redon et al. 2010; Stevens et al. 1992; Zhang et al. 2017). Cytokines IL13 and TNF-α, neutrophils, and COX2 damage DNA and increase mutations indirectly in IR cells (Ameziane-El-Hassani et al. 2015; Bisht et al. 2003; Burr et al. 2010; Hosseinimehr et al. 2015; Jackson et al. 1989; Ozyurt et al. 2014; Yan et al. 2006), and NO, TGF-β, NF-kβ, and COX2 increase DNA damage and mutations in bystander cells (Chai et al. 2013b; Dickey et al. 2009; Han et al. 2010; Redon et al. 2010; Shao et al. 2008; Zhou et al. 2005; Zhou et al. 2008). The DNA damage and mutations are reduced by blocking the inflammatory factors NF-kβ, COX2, TNF-α, TGF-β, or NO or with antioxidants (Burr et al. 2010; Han et al. 2010; Jackson et al. 1989; Rastogi et al. 2012; Zhang et al. 2017; Zhou et al. 2005, 2008). Inhibiting TNF-α or COX2 or using antioxidants also reduces GI after IR (Lorimore et al. 2008, 2011; Moore et al. 2005; Mukherjee et al. 2012; Natarajan et al. 2007).

Inflammatory pathways activated by IR promote tumor growth and metastasis. Exposure to IR or RONS sensitizes mammary epithelial cells to respond to TGF-β—which is widely activated by IR (Ehrhart et al. 1997). IR and TGF-β signaling leads to an epithelial-to-mesenchymal (EMT)-like transition, which disrupts the expression and distribution of cell adhesion molecules and multicellular organization and promotes invasion (Andarawewa et al. 2011, 2007; Iizuka et al. 2017; Park et al. 2003). This mechanism resembles wound healing (Koh and DiPietro 2011; Landen et al. 2016; Perez et al. 2014), but also resembles malignancy—invasive breast cancer cell lines overexpress TGF-β and respond to TGF-β with increased invasion (Gomes et al. 2012; Kim et al. 2004). The TGF-β-mediated response to IR likely involves an increase in senescence in fibroblasts (Liakou et al. 2016; Perrott et al. 2017; Sourisseau et al. 2011; Tsai et al. 2005).

IL6 is implicated in the carcinogenic response to IR. IL6 is expressed in mouse mammary gland after IR (Bouchard et al. 2013) and is produced by IR-senescent fibroblasts, but may also be expressed by epithelial cells after IR, since primary human mammospheres and pre-malignant mammary epithelial cell lines respond to IL6 with increased IL6 expression (Iliopoulos et al. 2009; Sansone et al. 2007). IL6 promotes the mobility and tumorigenesis of normal and breast cancer epithelial cells (Iliopoulos et al. 2009, 2010; Sansone et al. 2007; Sasser et al. 2007; Studebaker et al. 2008) through a NOTCH dependent pathway. NOTCH supports the renewal of stem-like cell populations (Sansone et al. 2007) and is implicated in the proliferative response to IR in mammary epithelia (Marusyk et al. 2014; Nguyen et al. 2011b; Tang et al. 2014). Interestingly, IL6 is also required for the growth and tumor promoting effects of breast cancer fibroblasts and fibroblasts from common sites of breast cancer metastasis (bone, lung) on ER-positive cancer cells in vitro and in vivo. ER-negative breast epithelial cells release autocrine IL6 and may, therefore, be less dependent on IL6 from fibroblasts, although IL6 also transforms these cells (Iliopoulos et al. 2009; Sasser et al. 2007; Studebaker et al. 2008).

### Data gaps and uncertainties

The effects of inflammation can be both pro- and anti-tumorigenic. For example, in addition to TGF-β’s role in EMT, in mammary epithelial cells TGF-β is essential to apoptosis of DNA damaged cells including IR-induced damage (Ewan et al. 2002), thus limiting GI (Maxwell et al. 2008). Inflammatory factors TNF-α and COX2 play a similar role in bone marrow of C57BL/6 mice (Lorimore et al. 2013). By eliminating cells with severe DNA damage and curtailing GI, apoptosis (and therefore TGF-β or TNF-α) limits the appearance of major (possibly carcinogenic) mutations following ionizing radiation. However, apoptosis (and thus TGF-β or TNF-α) can indirectly promote tumorigenesis through compensatory proliferation (Fogarty and Bergmann 2017; Loree et al. 2006).

Genetic background also influences the interaction between inflammation and tumorigenesis. Polymorphisms in inflammatory genes influence susceptibility to intestinal cancer following IR (Elahi et al. 2009). In the SPRET outbred mouse, higher baseline TGF-β during development decreases mammary tumor incidence following lower doses of IR (0.1 Gy), possibly by reducing ductal branching and susceptibility (Zhang et al. 2015). Conversely, the BALB/c mouse susceptible to mammary tumors after IR has a lower baseline TGF-β (and a polymorphism in a DNA damage repair-related gene). Early (4 h) after low dose (0.075 Gy), IR BALB/c mice have suppressed immune pathways and macrophage response but increased IL6, COX2, and TGF-β pathway activation in mammary gland compared to the tumor-resistant C57BL/6 mouse (Bouchard et al. 2013; Snijders et al. 2012). By 1 week after IR, the BALB/c mice show TGF-β-dependent inflammation in the mammary gland, and by 1 month after IR, their mammary glands show proliferation (Nguyen et al. 2011a; Snijders et al. 2012), suggesting that TGF-β is associated with inflammation, proliferation, and mammary tumorigenesis in these mice. Consistent with this pattern, BALB/c mice that are heterozygous for TGF-β are more resistant to mammary tumorigenesis following IR (Nguyen et al. 2011b). However, the BALB/c mouse also has a polymorphism in a DNA repair gene associated with IR-induced GI (Yu et al. 2001), making it difficult to distinguish potentially overlapping mechanisms.

While inflammatory signals are associated with bystander effects including DNA damage, GI, and mutation, these effects vary between organs in vivo (Chai et al. 2013a, b), by genotype (Coates et al. 2008; Lorimore et al. 2008, 2011), and by cell type (Chai et al. 2013a). Further research will be required to identify all the underlying factors determining differences in bystander effects, but one variable is the appearance of a protective apoptotic response to cytokines under some conditions (Lorimore et al. 2011, 2013).

One major piece of conflicting evidence comes from a direct test of the essentiality of inflammation to IR-induced carcinogenesis. In a mouse model of lymphoma, a mutation preventing the PIDD/NEMO-dependent activation of NF-kB blocks the early IR-induced activation of NF-kB (4–24 h) and production of TNF-α (5–48 h) but not lymphoma, suggesting that activation of these inflammatory factors is not essential in this time period (Bock et al. 2013). However, this study examined only day 1 post-IR time points for NF-kB activity, and did not block the production of IL6. Later activation of NF-kB or activation of other inflammation-related factors including IL6 and TGF-β could, therefore, have contributed to lymphoma.

## Epigenetic changes (KE)

### Evidence of activation by IR

IR produces epigenetic effects including changes in global and gene-specific DNA methylation, histone modification, and expression of methylation-related proteins and miRNA (Cui et al. 2011; Nzabarushimana et al. 2014; Prior et al. 2016; Raiche et al. 2004; Tawa et al. 1998) (see Supplemental Table 1 for more). Epigenetic effects appear in a variety of models within hours after IR and have also been detected days or weeks later (Chaudhry and Omaruddin 2012; Lima et al. 2014; Nzabarushimana et al. 2014; Pogribny et al. 2005).

The mechanism by which IR alters epigenetics is not well established, but RONS are implicated (Bernal et al. 2013; Galligan et al. 2014; Kloypan et al. 2015; Simone et al. 2009; Yang et al. 2014), and DNA damage is suggested (Koturbash et al. 2005, 2006b, 2008, 2016; Nishida et al. 2013; Pogribny et al. 2004, 2005; Szatmari et al. 2017).

Epigenetic changes vary widely with tissue and cell type, target, time after exposure, and sex (Lima et al. 2014; Miousse et al. 2017, 2014; Prior et al. 2016; Raiche et al. 2004), as well as between experiments (e.g., (Pogribny et al. 2004) vs (Raiche et al. 2004), (Miousse et al. 2014) vs (Miousse et al. 2017)), and do not necessarily correlate with expected changes in gene expression (Lima et al. 2014; Luzhna et al. 2015; Miousse et al. 2017, 2014).

Given the variation in epigenetic response in different tissues and sexes, studies in tissue other than female breast and mammary gland may not be informative for this AOP. However, a few studies have examined IR in breast or mammary gland or cells. IR of mammary gland in vivo causes global hypomethylation in the first 4 days and 6 weeks after 3–5 Gy IR (Kutanzi and Kovalchuk 2013; Loree et al. 2006) and hypomethylation of LINE1 in the 4 days after 0.1–2.5 Gy IR (Luzhna et al. 2015). In vitro, IR dose-dependently increased tumor suppressor miR-34a (only miR-34a, let-7a, let-21, and miR-21 were examined) in immortal MCF-10 cells but not MCF-7 cancer cells (Stankevicins et al. 2013). These studies suggest a possible epigenetic response to IR in breast and mammary gland, but the small number of studies and doses prevent drawing conclusions.

### Essentiality of epigenetic changes

**Essentiality is low.** Epigenetic changes in mRNA or miRNA contribute to the short-term DNA-damaging effect of IR on bystander cells. miRNA-containing vesicles from IR-exposed animals promote new DNA damage in unexposed animals (Szatmari et al. 2017) and RNase blocks the short-term DNA-damaging effects of exosome containing media from IR-exposed cells (Al-Mayah et al. 2012, 2015). MiRNA-21 is of particular interest: miRNA-21 in exosomes from exposed cells, miRNA-21 expressing cells, or bystander cells increases DNA damage in bystander cells, while miRNA-21 inhibitors reduce DNA damage from IR in bystander cells (Tian et al. 2015; Xu et al. 2015).

Several studies suggest that epigenetic changes also contribute to GI after IR. In one experiment, DNMTs (which methylate DNA) appear to be essential to transmission of GI to bystander cells in vitro (Rugo et al. 2011). Global hypomethylation is correlated with chromosomal aberrations in nuclear workers (Lee et al. 2015) and global hypomethylation associated with decreased DNMTs precedes DSBs and chromosomal rearrangements in vivo (Gaudet et al. 2003; Koturbash et al. 2006a). In cell lines with and without GI following IR exposure, the unstable cells had significant changes in repeat element methylation and in mRNA and miRNA associated with instability and mitochondrial respiration (Baulch et al. 2014; Thomas et al. 2012).

Epigenetic mechanisms including miRNAs, DNA, and histone methylation and DNMT expression are also implicated in tumorigenesis and invasion, with several studies in breast and mammary tissue. Invasive breast cancer tissue has more miRNA-21, and mammary adenocarcinomas from IR-exposed rats has enriched areas of both hyper and hypomethylation (Daino et al. 2018; Huang et al. 2009). Most of the genes that were differently methylated and expressed in exposed rats were related to developmental targets of PRC2, a histone methyltransferase (Daino et al. 2018). Mice under-expressing DNMT1 have global hypomethylation preceding thymic tumors (Gaudet et al. 2003), and more malignant breast cancer cells have more extensive hypomethylation and more changes in DNMT and histone methylation (Tryndyak et al. 2006).

### Data gaps and uncertainties

The evidence supporting a key role for epigenetic changes in breast cancer from IR is limited in three key areas. First, many studies have established that epigenetic effects vary greatly between organs, cell types, and sex (as well as with the other variables like time after exposure) (Lima et al. 2014; Miousse et al. 2014, 2017; Prior et al. 2016; Raiche et al. 2004). However, only eight studies in breast or mammary gland link IR with epigenetic changes or epigenetic changes with DNA damage or GI, and four of these use breast cancer cells. Given the variation between tissues, additional evidence in the breast or mammary gland is needed. Second, several studies report no link between epigenetic changes and changes in protein expression (Lima et al. 2014; Luzhna et al. 2015; Miousse et al. 2014, 2017), so this link needs to be verified or alternate mechanisms must be identified to assert a mechanistic effect. Finally, the large number of possible epigenetic changes, targets, and outcomes contributes to a general lack of understanding of the downstream effect of observed epigenetic changes.

Additional limitations and conflicting results also undermine confidence in the essentiality of this key event. Limited studies in vivo link epigenetic changes to DNA damage or GI. Furthermore, not all epigenetic changes necessarily increase DNA damage—instead, hypermethylation may protect against DNA damage. Cells exposed to repeated low (0.035 Gy) doses of IR show global hypermethylation but no increase in micronuclei, and blocking hypermethylation reduces the protective effect of low doses against DNA damage from higher (2 Gy) doses (Ye et al. 2013). Epigenetic changes including global methylation, repeat elements (including LINE1) and miRNA can also be seen in the absence of GI (Aypar et al. 2011; Miousse et al. 2014), and global methylation was not associated with GI in unstable cells after IR (Baulch et al. 2014).

## Key event measurement

Measurement of these key events is critical both to validate their roles in these and other AOPs, and to test agents for their ability to contribute to these key events, most of which are themselves adverse on the basis of their regulatory relevance. Available methods to measure these events are discussed below and summarized in Table 1. Standardized OECD guideline assays measure several key events and adverse outcomes, and limitations to those are noted where applicable. We also describe non-guideline methods to measure key events. Standardization and adoption into OECD-accepted guideline methods would advance the use of these assays and integration into chemicals testing. Very few of the assays are conducted in breast tissue, so additional validation is needed to ensure that results in other models can be generalized to breast.

There are several guideline assays for measuring breast cancer, DNA damage and mutation, and multiple non-guideline methods for measuring RONS, proliferation, inflammation, and epigenetic changes.

At least one method described for each key event has been applied in mammary glands or mammary cells. However, many of the assays specify a non-mammary tissue and even tissue-agnostic assays are not typically conducted in breast/mammary tissue. For chemical testing work in vitro, it is a priority to develop culture models that reflect the behavior of normal tissue (as opposed to cancer cells), and to establish the relevance of chosen models to the outcome of interest. For some key events, it may be important that models include multiple cell types (Morgan et al. 2020).

Non-guideline methods should be standardized for consistency. In particular GI, proliferation, epigenetic changes, and chronic inflammation all have a strong temporal component, and methods should be developed to measure these key events at multiple time points along with the other standard procedures to improve reproducibility.

### Breast cancer

The 2-year rodent carcinogenicity bioassay is the primary assay for breast cancer (Rudel et al. 2007). The assay is included in the OECD Test No. 451 and 453 for carcinogenicity and combined toxicity and carcinogenicity (OECD 2009a, b), and is also used by the US National Toxicology Program (NTP) (Chhabra et al. 1990), and the FDA (2007), and referenced by the EPA (EPA 2005) in guidelines for risk assessments. Other assays from short term (2–4 weeks) and subchronic (90 day) to chronic (1 year) toxicity also call for the documentation of mammary tumors (FDA 2007; OECD 2018), so these assays could capture the early onset of tumors if the methods were sufficiently sensitive to detect small lesions (Makris 2011), and could be modified to report earlier key events like hyperplasia and inflammation. For example, evaluation of breast tissue for hyperplasia or proliferative epithelial lesions could be done at several early time points and could trigger later evaluations. Doing these observations after BrdU injection and staining of tissue sections to evaluate proliferation could also increase sensitivity of the assay.

Several characteristics of classic cancer bioassays limit the sensitivity of these assays to mammary gland carcinogens. First, assays do not require prenatal or early post-natal exposures for carcinogenicity testing. The NTP often starts exposures at 5–6 weeks of age and OECD regulatory assay exposures suggest (but do not require) exposures beginning after weaning and before 8 weeks of age. Since sensitivity appears to peak around or before week 7 for these DNA-damaging agents (around puberty) (Imaoka et al. 2013), studies that start dosing later may have reduced sensitivity. Also, assays that initiate exposure in mature animals have diminished sensitivity to hormonally active agents that act during development to alter breast development and increase future susceptibility to cancer, such as estrogenic hormones, DDT, and dioxins (EPA 2005; Rudel et al. 2011). Second, carcinogenicity assay guidelines do not require the best methods for detecting tumors in mammary gland: whole mount preparations of mammary gland coupled with longitudinal sections (dorsoventral sections parallel to the body) of mammary gland for histology (Tucker et al. 2017). Palpation and transverse sections of mammary gland can easily miss tumors or lesions of interest. The NTP does specify these preferable methods for mammary gland analysis in reproductive toxicity guidelines (NTP 2011a) and an NTP workgroup recommends the early life and in utero dosing for cancer bioassays (Thayer and Foster 2007).

Two additional limitations may reduce the sensitivity of standard carcinogenicity assays. First, benign tumors are not always interpreted as an indicator of carcinogenicity, leading to a possible underestimation of risk. NTP and EPA guidance suggest that benign tumors provide additional weight of evidence if malignant tumors are also present or if studies suggest benign tumors can progress to carcinogenicity. In a short-term study, proliferative epithelial lesions, hyperplasia, or benign tumors may indicate a need for a longer term study. Benign mammary tumors (fibroadenomas) almost always coincide with carcinogenic tumors in mammary gland or other organs, and carcinomas sometimes grow from fibroadenomas (Rudel et al. 2007; Russo 2015), suggesting that benign tumors are an underutilized indicator of mammary carcinogenicity.

Finally, the dose-selection guidance in carcinogenicity testing typically calls for a high dose that is sufficiently toxic to suppress body weight (OECD 2009a). However, body weight in rodent cancer bioassays is correlated with mammary tumors, and so, toxicity-induced weight loss at higher doses may result in fewer mammary tumors, and as a result, observed mammary tumors at the lower doses may be dismissed because of the apparent lack of a dose–response (Haseman et al. 1997; Rudel et al. 2007). This potential for suppression of mammary tumors by toxicity at high dose should be considered in weight of evidence determinations for carcinogenicity.

### RONS

RONS is typically measured using fluorescent or other probes that react with RONS to change state, or by measuring the redox state of proteins or DNA (Dickinson and Chang 2011; Griendling et al. 2016; Wang et al. 2013). Optimal methods for RONS detection have high sensitivity, selectivity, and spatiotemporal resolution to distinguish transient and localized activity, but most methods lack one or more of these parameters.

Molecular probes that indicate the presence of RONS species vary in specificity and kinetics (Dickinson and Chang 2011; Griendling et al. 2016; Wang et al. 2013). Small molecule fluorescent probes can be applied to any tissue in vitro, but cannot be finely targeted to different cellular compartments. The non-selective probe DCHF was widely used in the past, but can produce false-positive signals and may not be optimal (Griendling et al. 2016). EPR (electron paramagnetic resonance spectroscopy) provides the most direct and specific detection of free radicals, but requires specialized equipment. Fluorescent protein-based probes can be genetically engineered, expressed in vivo, and targeted to cellular compartments and specific cells. However, these probes are very sensitive to pH in the physiological range and must be carefully controlled. Newer selective small molecule probes such as boronate-based molecules are being developed, but are not yet widely used. EPA’s ToxCast series of high-throughput in vitro assays (Thomas et al. 2019) includes an assay that measures mitochondrial membrane potential in combination with dihydroethidium, a qualitative measure of superoxide formation (Giuliano et al. 2010; Kalyanaraman et al. 2012).

Alternative methods involve the detection of redox-dependent changes to cellular constituents such as proteins, DNA, lipids, or glutathione (Dickinson and Chang 2011; Griendling et al. 2016; Wang et al. 2013). However, these methods cannot generally distinguish between the oxidative species behind the changes, and cannot provide good resolution for kinetics of oxidative activity.

These methods are readily applied to mammary cells. However, measurements in different cell types may vary based on differences in the expression of endogenous antioxidants (Kannan et al. 2014). A standard comparison of response across cell types would be useful.

### DNA damage, GI, and mutation

DNA damage can be studied in isolated DNA, fixed cells, or living cells. Types of damage that can be detected include single- and double-strand breaks, nucleotide damage, complex damage, and chromosomal or telomere damage. The OECD test guideline for DNA synthesis Test No. 486 (OECD 1997b) detects nucleotide excision repair, so it will reflect the formation of bulky DNA adducts but not the majority of oxidative damage to nucleotides, which is typically repaired via the Base Excision Repair pathway. This test is not recommended by some agencies, because it is not generalizable beyond the liver (EFSA Scientific Committee et al. 2017). The OECD test guideline alkaline comet assay Test No. 489 (OECD 2016f) detects single- and double-strand breaks, including those arising from repair as well as some (alkali sensitive) nucleotide lesions including some lesions from oxidative damage. OECD tests for chromosomal damage and micronuclei Test Nos. 473, 475, 483, and 487 measure longer term effects of DNA damage, but these tests require the damaged cell to subsequently undergo replication (OECD 2016a, b, d, e). They can, therefore, reflect a wider range of sources of DNA damage, including changes in mitosis. While the comet assay test 489 does not specify a target tissue, it is not typically performed in mammary cells (OECD 2016f) and the other guideline tests are never performed in mammary cells or tissues. Although Rube 2008 reports no difference in degree of DNA damage and repair kinetics in five tissues, expression of DNA repair proteins can vary between tissues (Gottlieb et al. 1997; Gurley and Kemp 2007; Sun et al. 2019) which could lead to a difference in damage observed at various time points. For genotoxicity testing, variations in transport and metabolism can also lead to differences between mammary gland and other tissues (Ding et al. 2014). These tests should, therefore, be generally validated for or performed in mammary gland to address risk in this tissue.

Many other (non-test guideline) techniques have been used to examine specific forms of DNA damage. The comet chip facilitates high-throughput comet assays (Ge et al. 2014). Double-strand breaks are commonly measured microscopically using fluorescently labeled antibodies to H2AX or other labeled probes because of the significant risk attributed to breaks and the relative ease of detecting and quantifying them (Lorat et al. 2016; Nikitaki et al. 2016; Ojima et al. 2008; Rothkamm and Lobrich 2003). Measurement of single-strand break repair is less common but possible by labeling single-strand break repair protein XRCC2 (Lorat et al. 2016; Nikitaki et al. 2016). Base lesions can also be detected using labeled probes for base excision repair enzymes, or by chemical methods such as mass spectroscopy (Madugundu et al. 2014; Nikitaki et al. 2016; Ogawa et al. 2003; Ravanat et al. 2014). Refinements on these methods characterize complex or clustered damage, in which various forms of damage occur in close proximity on a DNA molecule (Lorat et al. 2016; Nikitaki et al. 2016). Some DNA-damaging agents act by directly binding to DNA to form adducts, the detection of which is indicative of the potential for mutation (Rundle 2006). EPA’s ToxCast uses a labeled antibody to p53 to indicate non-specific increases in DNA damage and stress (Giuliano et al. 2010). These methods can be or have been applied to mammary or breast cells (Al-Mayah et al. 2012, 2015; Dutta et al. 2014; Haegele et al. 1998; Hernandez et al. 2013; Jones et al. 2007; Kirshner et al. 2006; Redon et al. 2010; Snijders et al. 2012; Soler et al. 2009; Wang et al. 2015). Since results in other tissue may or may not be applicable to breast tissue, they should be performed in breast to best inform risk of breast cancer.

Certain challenges are common to all methods of detecting DNA damage. In the time required to initiate the detection method, some DNA may already be repaired, leading to undercounting of damage. On the other hand, apoptotic DSBs may be incorrectly included in a measurement of direct (non-apoptotic) induction of DSB damage unless controlled. All methods have difficulty distinguishing individual components of clustered lesions, and microscopic methods may undercount disparate breaks that are processed together in repair centers (Barnard et al. 2013). Methods that use isolated DNA (gel electrophoresis and analytical chemistry) are vulnerable to artifacts and must ensure that the DNA sample is protected from oxidative damage during extraction (Barnard et al. 2013; Pernot et al. 2012; Ravanat et al. 2014).

Finally, tests for mutations reveal past DNA damage that result in a heritable change, including multiple guideline tests (OECD Ames Test No. 471 (OECD 1997a), Hprt or Xprt Test No. 476 (OECD 2016c), Transgenic Rodent Somatic and Germ Cell Gene Mutation Assays Test No. 488 (OECD 2013; White et al. 2019), and Thymidine kinase Test No. 490 (OECD 2016g)) and other assays such as the RaDR-GFP transgenic mouse that detects mutational errors in homologous recombination (Kiraly et al. 2015; Sukup-Jackson et al. 2014). One of the approved TG 488 transgenic mouse lines (Jakubczak et al. 1996) has been used to measure mutations in mammary gland in vivo, and the RaDR-GFP mouse and several TG 488 approved lines have been used for primary or established mammary cell lines (Sukup-Jackson et al. 2014; White et al. 2019). The other tests are limited to specific non-mammary tissues, and should be validated for relevance to mammary gland.

No validated protocols exist specifically to measure GI, but a wide range of the above methods are currently performed at various times after exposure to verify ongoing damage or mutations, including in mammary gland or breast cells (Al-Mayah et al. 2012, 2015; Jakubczak et al. 1996; Maxwell et al. 2008; Ponnaiya et al. 1997a, b; Snijders et al. 2012; Ullrich and Davis 1999; Yu et al. 2001). Since GI can be expressed in a variety of ways, a validated protocol should be developed that measures multiple outcomes and time points.

### Proliferation and hyperplasia

Past cellular proliferation can be measured directly using labels that are incorporated into cells upon cell division (BRDU or cytoplasmic proliferation dyes) or indirectly by measuring a change in population size. Ongoing proliferation can be quantified by labeling a protein associated with the cell cycle (e.g., Ki67). Methods have been recently reviewed (Menyhart et al. 2016; Romar et al. 2016) and histopathological assessments for mammary gland hyperplasia in in vivo guideline toxicity studies are reviewed in (Makris 2011).

For in vitro assays, EPA’s ToxCast has a BRDU assay for proliferation in HepG2 (Giuliano et al. 2010) and an assay for estrogen-mediated proliferation in T47D breast cancer cells (Judson et al. 2015). Since many of the cells are proliferating in the sub-confluent cell-culture system, it is not clear how sensitive the assay is to increased proliferation. A large body of work has demonstrated that cancerous and non-cancerous cells grow differently in 3D culture systems and that the differences are not apparent in 2D. Furthermore, the effects of chemical exposure on cell growth and development may only be manifest in tissue that contains multiple cell types together. For example, mammary pre-adipocytes produce estradiol via aromatase and epithelial cells proliferate in response (Morgan et al. 2020). Finally, while many toxicity testing models use cancer cell lines, non-cancerous cells may respond differently. Thus, there is considerable need for investment to strengthen in vitro models.

Hyperplasia is measured histologically based on increased cell numbers leading to increased layers of cells and tissue depth (Collins 2018). Several modifications to guideline in vivo toxicity studies would enhance the detection of hyperplasia and could also detect altered mammary development. These include adding time points and more detailed protocols for evaluating mammary tissue, assessment in male as well as female mammary tissue, longitudinal rather than transverse sectioning, and BrDU injection and staining to better detect proliferation. These assessments could be added to 90-day, 28-day, and 14-day studies, and to studies with developmental exposure such as EPA’s pubertal and the OECD one-generation reproduction study (Makris 2011; Rudel et al. 2011).

In mammary gland, Ki67 or histology is commonly used to characterize proliferation both in vivo and in vitro, and other methods can be easily applied as well. Given the potential variation in endogenous and context-dependent proliferation between mammary and other tissues, findings in non-mammary tissues should be either validated or measured directly in mammary tissues.

### Inflammation

Inflammation is commonly measured using cytokine mRNA or extracellular concentrations of cytokines like IL-6, expression of proteins including COX2 or iNOS, activation of key inflammatory signaling molecules MAP kinases and transcription factors NF-kB, and AP1, as well as histological measures like leukocyte infiltration.

Typical assays to detect changes in protein concentration, phosphorylation, or localization include ELISA (El-Saghire et al. 2013; Partridge et al. 2010; Siva et al. 2014), Western Blot (Chai et al. 2013b; Ha et al. 2010), electrophoretic mobility shift assay or EMSA (Haase et al. 2003), or immunostaining (Chai et al. 2013b; Wang et al. 2015). Immunostaining can also detect infiltration of immune cells, a marker for inflammation (Ebrahimian et al. 2018; Monceau et al. 2013; Moravan et al. 2011). Changes in gene transcription are detected using PCR (Azimzadeh et al. 2011; Bouchet et al. 2013; Moravan et al. 2011; Snijders et al. 2012; Wang et al. 2014b). Other methods include histopathological examination of tissue for indicators of inflammation (Haddadi et al. 2017), or measurements of leukocyte adhesion (Arenas et al. 2006; Rodel et al. 2008).

EPA’s ToxCast has a range of assays measuring inflammation-related outcomes (Houck et al. 2009). All of these assays stimulate cells with inflammatory stimuli in addition to the test reagent, so it is possible (though not so reported) that these are more sensitive to reduction than induction of inflammatory pathways.

No specific test is recommended for chronic inflammation. Like GI, the above assays may be applied at various time points after exposure as an indicator of chronic response. We support the development of methods optimized for chronic inflammation. These methods would likely require long-term or repeated exposures. Additionally, since inflammation is a tissue response and not a cell response alone, such an assay would ideally capture tissue interactions.

The above methods are readily applied to mammary tissue (Barcellos-Hoff et al. 1994; Bouchard et al. 2013; Datta et al. 2012a; Snijders et al. 2012; Wang et al. 2015). Since it is not well established how inflammatory responses in a different tissue might apply to mammary tissue, validation studies should be conducted to establish the tissue variation in assay responses to inflammatory stressors, or studies should be conducted in mammary gland.

### Epigenetic changes

Epigenetic changes occur and are measured in several different ways. Shifts in global methylation are measured using the [3H]dCTP extension assay (Koturbash et al. 2016), HPLC for 5mdC (Wang et al. 2014a), or quantitative immunoassay for 5-mc (Nzabarushimana et al. 2014). Gene-specific changes in methylation can be measured using MeDIP-on-chip (Hsu et al. 2015), methylation-sensitive/dependent restriction enzymes and qPCR (Nzabarushimana et al. 2014; Oakes et al. 2006a) and bisulfite sequencing and PCR (Wang et al. 2014a). Changes in miRNA expression are measured using qRT-PCR (Stankevicins et al. 2013; Szatmari et al. 2017), hybridization (e.g., microarray, etc.) (Aypar et al. 2011; Jacob et al. 2013), and sequencing (Mestdagh et al. 2014), while histone methylation is measured using ChIP (Prior et al. 2016) or immunohistochemistry (Kutanzi and Kovalchuk 2013).

The most consequential outcome of epigenetic changes is changes in gene expression, which can be measured using western blot (Wang et al. 2014a), qRT-PCR (Prior et al. 2016), or whole transcriptomics, e.g., RNA microarray and RNA-seq (Hrdlickova et al. 2017; Manzoni et al. 2018).

These methods can be and have been applied to breast and mammary gland (Kutanzi and Kovalchuk 2013; Loree et al. 2006; Luzhna et al. 2015; Stankevicins et al. 2013). Considering the tissue, sex, and temporal variability reported in epigenetic effects, experiments that differ in one of these contexts should not be extrapolated to mammary gland and instead the study should directly examine mammary tissue.

## Weight of evidence for essentiality of key events

The evidence was evaluated for the essentiality of each key event to downstream events and to breast cancer and is summarized in Table 2. IR appears to be a “complete” carcinogen in the mammary gland in that it acts as an initiator through the formation of oxidative stress and pro-mutagenic DNA damage (the MIEs) and as a promoter through increasing inflammation and proliferation, similar to many chemical carcinogens (Russo and Russo 1996). We have high confidence in the evidence linking stressor (IR) with adverse outcome (breast cancer). The weight of evidence for breast carcinogenesis arising from RONS and DNA damage leading to GI, mutation, proliferation, and hyperplasia is high, while the weight of evidence for the second pathway from RONS and DNA damage to GI, inflammation, proliferation, hyperplasia, and breast cancer is moderate based on the moderate confidence in the inflammation key events. The weight of evidence for epigenetics in these pathways is low based on the current evidence.

**Table 2:** Weight of evidence for essentiality of key events to the breast cancer pathway

Key events	Essentiality
Increase in reactive oxygen and nitrogen species (RONS)	**Essentiality is high**. A large number of studies using antioxidants or other interventions to reduce RONS show a reduction in DNA damage, GI, mutations, and inflammation. Additional support comes from experiments increasing external oxidants like H_2_O_2_, which show that RONS are independently capable of causing DNA damage, mutations inflammation, and epigenetic changes. Uncertainties arise from the smaller effects of RONS on DNA damage compared with ionizing radiation. Mammary gland relevance is less certain due to the relatively few experiments in breast tissue
Increase in DNA damage, GI, and mutation	**Essentiality is high.** The increase in DNA damage and GI precedes mutations, proliferation, and tumorigenesis, while antioxidants that reduce DNA damage and GI also reduce mutations and chromosomal damage. Knock-out and knock-in experiments show that mutations in certain key genes increase tumorigenesis. However, an ongoing debate fueled by transplant studies that show the importance of tissue environment for tumorigenesis questions the necessity of mutations as the singular driver of tumorigenesis, since mutation also follows the other tumor-promoting events like inflammation and proliferation
Increase in Proliferation and Hyperplasia	**Essentiality is high.** Evidence comes from transplant experiments showing that non-proliferating tissue is less tumorigenic than proliferating lesions, and from interventions that reduce both proliferation and tumors. Further evidence comes from animals that are resistant to both mammary gland proliferation and tumors from ionizing radiation. Uncertainty arises from conflicting evidence on the tumorigenicity of hyperplasia, and the absence of hyperplasia observed before some tumors
Increase in inflammation	**Essentiality is moderate.** Evidence comes from using genetic modifications, antibodies, and antioxidants to reduce inflammatory and anti-inflammatory factors. These interventions reduce DNA damage, mutations, and mechanisms contributing to tumorigenesis and invasion. Uncertainty arises from conflicting effects in different genetic backgrounds and in different organs, and the pro-apoptotic effect of TGF-β
Epigenetic changes	**Essentiality is low.** The strongest evidence shows that blocking miRNAs and DNMTs reduces bystander DNA damage and GI. The remainder of the evidence correlates epigenetic changes with downstream effects. Uncertainty arises from the tissue dependence of epigenetic effects combined with the low number of studies in mammary gland, and from the discontinuity between epigenetic changes and changes in gene and protein expression

## Discussion

### Context

DNA damage and mutation events correspond to the classical mechanism of carcinogens as mutagens first identified over 50 years ago, while the role of RONS and inflammation is in alignment with the tissue-oriented field theory which elevates the importance of tissue environment (Baker 2015). Modern conceptions for carcinogenesis marry these models and recognize that most carcinogens act on multiple biological targets. For example, IARC’s review of biological activity for known human carcinogens led to the listing of ten “characteristics of carcinogens” that include inducing genotoxicity, GI, oxidative stress, and chronic inflammation, among others (Smith et al. 2016), and the 2012 IARC monograph on carcinogenicity of ionizing radiation reflects many of the same key events which we have highlighted here (IARC 2012). The interaction of key events in this AOP with hormonally driven development and proliferation contributes additional complexity for breast and other hormonally mediated cancers. Because a single agent often has more than one of these characteristics, and host susceptibility and co-exposures also influence these pathways, a simple linear AOP will not adequately represent the underlying processes. For example, experiments suggest that the likelihood of mammary cancer after IR is lower when ovaries are removed and that both mutation and estrogen exposure (via effects on proliferation and other key events) contribute to breast carcinogenesis allowing for additive or synergistic effects (Broerse et al. 1987; Clifton et al. 1985; Cronkite et al. 1960).

### Dose–response and low-dose epidemiology and animal studies

Although this paper describes a qualitative AOP, an overview of dose–response observations from low-dose studies provides useful context.

Comprehensive and authoritative reviews of ionizing radiation consistently conclude that the linear no threshold dose–response (sometimes with slope adjustments for low dose or low-dose rate) is the most appropriate and protective model for cancers following exposure to IR (Ruhm et al. 2016; Shore et al. 2018). Low power limits precise estimations of the dose–response in people exposed to low doses (below approximately 0.1 Gy) of mixed or low LET IR like the atomic bomb or medical radiation (Grant et al. 2017; Ozasa et al. 2012; Ruhm et al. 2016; Shore et al. 2018; Suzuki and Yamashita 2012), but solid cancer dose–response in the atomic bomb Life Span Study (LSS) cohort is not significantly different from linear (Furukawa et al. 2016; Grant et al. 2017; Preston et al. 2007). Multiple epidemiological studies of specific cancer sites and total solid cancer following in utero and childhood exposure and in atomic bomb, nuclear worker, and population studies also support significant effects at low doses (Cohen et al. 2018; Little et al. 2014; Mathews et al. 2013; Meulepas et al. 2019; Pearce et al. 2012; Preston et al. 2007; Ruhm et al. 2016; Shore et al. 2017), although a metanalysis of these studies reports that IR does pose a greater cancer risk per unit dose at high-dose rates than at low-dose rates (less than 0.0001 Gy/minute) (Shore et al. 2017).

Although total solid cancers have a linear dose–response, dose–response shape differs by tumor site and sex (Grant et al. 2017; Ozasa et al. 2012; Shore et al. 2018; Suzuki and Yamashita 2012). In males, total tumors and some specific tumor sites have upwardly curving dose–responses with shallower dose–response at low vs high doses, while total tumors in females and sex-specific tumors (combined across sexes) have linear dose–responses (Grant et al. 2017; Sasaki et al. 2014). Studies in animals support overall linearity and show variation in dose–response between affected tissues (Little 2018; Tran and Little 2017).

Available data suggest that breast cancer dose–response is linear. A pooled analysis of eight breast cancer cohorts found no evidence to contradict linear dose–response for breast cancer, although they did find lower excess risk for low-dose rates (Preston et al. 2002). Cancer data from women exposed as infants (mean dose 0.186 Gy) show a linear increased risk of breast cancer (Eidemuller et al. 2015). More recent examinations of LSS data using updated dose estimates support this conclusion, finding no significant non-linearity of breast cancer over the whole or 0–2 Gy dose range (Preston et al. 2007), but the most recent dose and incident LSS data have not yet been reported for breast cancer.

Like breast cancer in people, the dose–response for mammary gland tumors in rodents is linear (Gragtmans et al. 1984; Imaoka et al. 2013), but has not been widely investigated in the low-dose range (< 0.1 Gy). One low-dose experiment reporting mammary tumors did not find a significant change (increase or decrease) in mammary tumors at chronic low doses, all of which were below 0.021 Gy/day (Tanaka et al. 2007). Although each group contained 500 animals, this study was performed in B6C3F1 mice which do not carry the mouse mammary tumor virus and are not otherwise particularly susceptible to mammary tumors, so the power to detect changes may still have been insufficient (Bennett and Davis 2002).

Animal and in vitro studies suggest that high LET radiation (alpha particles from cosmic radiation and radioactive decay of isotopes such as plutonium from nuclear materials or radon gas) has a linear or even supralinear dose–response at low doses (Azzam et al. 2016; Little 2018; Tran and Little 2017).

### Generalizability to other agents

Breast carcinogenesis from IR and other DNA-damaging agents has more similarities than differences (Imaoka et al. 2009). Both IR and other DNA-damaging agents form adenocarcinomas in rodents with similar pathology and gene expression, although IR also creates a much larger fraction of fibroadenomas than the other DNA-damaging agents (Imaoka et al. 2009). Carcinogenicity for IR and chemical mammary carcinogens NMU and DMBA varies consistently with age and exposure to ovarian hormones (Imaoka et al. 2009; Medina 2007; Russo 2015). Breast carcinogenesis from IR and chemical carcinogens depends strongly on developmental or ongoing exposure to ovarian hormones (Nandi et al. 1995; Russo 2015), and estrogen status of IR and chemical carcinogen-induced tumors increases with ovarian hormone exposure in rats (Imaoka et al. 2009; Nandi et al. 1995). The mammary gland is especially susceptible to both IR and mammary carcinogens DMBA and NMU around puberty. This is presumably because puberty is when undifferentiated cells are present in large numbers and poised to undergo subsequent proliferative expansion, although additional factors including metabolism and expression of DNA damage repair genes contribute to variations in the age of maximal susceptibility between agents (Imaoka et al. 2009, 2013, 2011; Medina 2007). IR has an additive effect in combination with NMU (Imaoka et al. 2014), consistent with general accepted risk assessment assumptions of additivity in carcinogenesis (National Research Council 2009). Some differences between mammary carcinogens appear around the protective role of breast maturation: pregnancy appears to be more protective in rats exposed to chemical carcinogens than in rats exposed to IR (Imaoka et al. 2009).

The role of DNA damage, mutation, and proliferation outlined in this AOP would presumably apply to other DNA-damaging agents, while the role of RONS and inflammation is more likely to vary between DNA-damaging and other agents based on their ability to induce these key events. DNA-damaging agents differ in the degree, type, and reparability of the DNA damage which they cause. Mammary carcinogens NMU, DMBA, PhIP, and urethane mostly cause adducts with single-nucleotide substitutions (Committee to Assess Health Risks from Exposure to Low Levels of Ionizing Radiation 2006; Imaoka et al. 2009; Nik-Zainal et al. 2015; Sherborne et al. 2015; Westcott et al. 2014). Like ionizing radiation, mammary carcinogen PhIP can cause amplifications and NMU can cause GI (Goepfert et al. 2007; Imaoka et al. 2009). While IR also induces adducts, it characteristically generates complex damage and double-strand breaks leading to deletions and inversions as well as amplification and GI (Behjati et al. 2016; Datta et al. 2012b; Mavragani et al. 2017; Mukherjee et al. 2012; Pazhanisamy et al. 2011; Snijders et al. 2012; Yang et al. 2015). The prevalence of complex damage and double-strand breaks is likely due to the density of damage delivered by ionizing radiation, but is also attributable to oxidative activity, since IR creates an oxidative state and H_2_O_2_ and other oxidizing agents can also cause complex damage, double-strand breaks and mutations (Cadet et al. 2017; Seager et al. 2012; Sharma et al. 2016). Radiomimetic compounds (used in chemotherapy) also cause double-strand breaks and simple complex damage. Agents like bleomycin cause double-strand breaks through oxidized lesions (Regulus et al. 2007), while agents like etoposide and cisplatin cause double-strand breaks by interfering with DNA replication forks (Kawashima et al. 2017).

Proliferation and inflammation are also implicated in chemical carcinogenicity. The aforementioned pubertal susceptibility to carcinogen exposure implies an important role for proliferation, as does the fact that tumorigenesis following NMU depends on proliferation during treatment (Medina 2007). Like IR, NMU and DMBA promote hyperplasia in terminal-end buds and ducts and ductal carcinoma in situ leading to carcinogenesis (Goepfert et al. 2007; Imaoka et al. 2009; Medina 2007; Russo 2015). In terms of inflammation, like IR, some chemical carcinogens increase inflammatory reactions in mammary stroma and also show a tumor-promoting effect of exposed stroma (Barcellos-Hoff and Ravani 2000; Maffini et al. 2004; Nguyen et al. 2011b; Russo and Russo 1996) and although bleomycin has not been characterized for its effects on mammary stroma or mammary carcinogenesis, it causes lung fibrosis (an inflammatory reaction) so consistently that it is used as a research model for that endpoint (Moeller et al. 2008).

### Other key events

This review does not address a large number of biological processes that would be expected to interact with the key events described here. For example, changes in the tissue microenvironment such as increased breast density are associated with increased risk of cancer. Multiple changes occur in the tissue microenvironment following IR and inflammation that may increase density, but insufficient data are available to characterize these changes as a separate key event after IR. Subsequent experiments should examine the time course, dose-dependence, and essentiality of changes to the breast microenvironment including breast density after IR and consider whether these factors should be considered a separate key event. Other important biological effects of IR that would be expected to interact with this pathway include immune surveillance which may change with the inflammatory environment after IR (Barcellos-Hoff 2013; Lumniczky and Safrany 2015; Schreiber et al. 2011); IR effect on survival/apoptosis and interactions of apoptosis with inflammation, mutation, compensatory proliferation, and selection process; changes to DNA repair or the many possible influences on those events. The interaction of these key events with hormonally driven development and proliferation represent a critical aspect of breast carcinogenesis with significant implications for sensitivity of experimental models as well as dose–response.

### Data gaps

Guideline tests are not established for important cancer-related endpoints such as RONS, GI, proliferation, hyperplasia, and inflammation. In addition, assessment of mammary gland effects in guideline studies is limited, and so, important changes can be missed (Makris 2011; Rudel et al. 2011). Adopting standardized methods for these key events and ensuring that the methods are either applied in mammary gland or that results in different tissues are relevant to mammary gland would better capture the potential of a chemical to act as a breast carcinogen. Improved assays are needed to measure genomic instability and chronic inflammation, as well as the interaction with hormonally driven development and proliferation.

An important gap relates to knowledge of the interactions between the key events in this AOP and hormonally driven development and proliferation in the breast, and this gap is a particular barrier to developing a quantitative AOP. Because stressors often activate multiple parts of this pathway, we caution against generalizing the dose–response across different stressors. We expect a different dose–response relationship depending on if a stressor only increases one part of the pathway (such as increasing mutations) or affects multiple parts (such as RONS, mutation and proliferation). In addition, individuals live in a complex mixture of environmental and genetic influences, some of which act on one or more events in the same carcinogenesis pathway to increase the overall risk of breast cancer. As a result, dose–response relationships for a particular stressor will also vary depending on host susceptibilities and co-exposures.

Evidence suggests that breast cancer increases linearly with dose even at lower doses, and that breast cancer incidence is elevated at doses relevant to diagnostic radiation (0.001 to 0.1 Gy), but greater power is needed to determine the precise shape of the breast cancer dose–response at these doses. DNA damage appears to increase linearly even at low doses, but even for this well-studied key event, there are insufficient studies with multiple low doses and sufficient power to enable the estimate of the lower end of the dose–response relationship in mammary gland. For other key events, a few studies inform the dose–response in any tissue. For example, although many studies support the link between IR, RONS, and DNA damage, a few RONS studies use mammary tissue, multiple doses, doses below 1 Gy, or explicitly measure RNS presenting an addressable data gap. Given these uncertainties, a better characterization of the low-dose end of the dose–response for the key events would give a more complete picture of the relative contribution of these different events and pathways to the breast cancer outcome across a range of doses. If more evidence is needed, it would be helpful to measure the dose–response of DNA damage and other key events in breast tissue using the highest possible power in this lower dose range and test multiple doses.

## Conclusions

This paper extends the characteristics of mammary carcinogens beyond DNA damage, highlighting the important role in breast cancer of agents that increase RONS, GI, cell proliferation, and inflammation. The interaction of these key events with hormonally driven development and proliferation represent a critical aspect of breast carcinogenesis. The AOPs which we have described for ionizing radiation leading to breast cancer are consistent with recent enumeration of characteristics of many known human carcinogens by IARC and other scientists (IARC 2012; Smith et al. 2016). Standardized assays are needed to measure RONS, GI, proliferation, and chronic inflammation, and breast cancer-related effects may be missed unless mammary gland is more consistently included in testing and assays in other tissues are evaluated to see if they can be generalized to breast. Adopting standardized methods for these key events would better capture the potential of a chemical to act as a breast carcinogen.

## Electronic supplementary material

Below is the link to the electronic supplementary material.

Supplementary file1 (XLSX 145 kb)

Supplementary file2 (XLSX 22 kb)

Supplementary file3 (XLSX 33 kb)

## Abbreviations

IR: Ionizing radiation
RONS: Reactive oxygen and nitrogen species
AOP: Adverse outcome pathway
